# The Effect of Sodium Bicarbonate, a Beneficial Adjuvant Molecule in Cystic Fibrosis, on Bronchial Epithelial Cells Expressing a Wild-Type or Mutant CFTR Channel

**DOI:** 10.3390/ijms21114024

**Published:** 2020-06-04

**Authors:** Ilona Gróf, Alexandra Bocsik, András Harazin, Ana Raquel Santa-Maria, Gaszton Vizsnyiczai, Lilla Barna, Lóránd Kiss, Gabriella Fűr, Zoltán Rakonczay, Rita Ambrus, Piroska Szabó-Révész, Fabien Gosselet, Pongsiri Jaikumpun, Hajnalka Szabó, Ákos Zsembery, Mária A. Deli

**Affiliations:** 1 Institute of Biophysics, Biological Research Centre, H-6726 Szeged, Hungary; grof.ilona@brc.hu (I.G.); bocsik.alexandra@brc.hu (A.B.); harazin.andras@brc.hu (A.H.); anaraquel.santamaria@brc.hu (A.R.S.-M.); vizsnyiczai.gaszton@brc.hu (G.V.); barna.lilla@brc.hu (L.B.); 2 Doctoral School of Biology, University of Szeged, H-6720 Szeged, Hungary; 3 Department of Pathophysiology, University of Szeged, H-6725 Szeged, Hungary; kiss.lorand@med.u-szeged.hu (L.K.); gabriella.fur@gmail.com (G.F.); rakonczay.zoltan@med.u-szeged.hu (Z.R.J.); 4 Institute of Pharmaceutical Technology and Regulatory Affairs, University of Szeged, H-6720 Szeged, Hungary; arita@pharm.u-szeged.hu (R.A.); revesz@pharm.u-szeged.hu (P S.-R.); 5 Blood-Brain Barrier Laboratory, UR 2465, Artois University, F-62300 Lens, France; fabien.gosselet@univ-artois.fr; 6 Department of Oral Biology, Semmelweis University, H-1089 Budapest, Hungary; jaikumpun@gmail.com (P.J.); zsembery.akos@dent.semmelweis-univ.hu (Á.Z.); 7 Department of Pediatrics, Fejér County Szent György University Teaching Hospital, H-8000 Székesfehérvár, Hungary; szabo65@yahoo.com

**Keywords:** bronchial epithelial cells, CFTR channel, cystic fibrosis, cytokine, impedance kinetics, permeability, sodium bicarbonate, tight junctions, transepithelial electric resistance

## Abstract

Clinical and experimental results with inhaled sodium bicarbonate as an adjuvant therapy in cystic fibrosis (CF) are promising due to its mucolytic and bacteriostatic properties, but its direct effect has not been studied on respiratory epithelial cells. Our aim was to establish and characterize co-culture models of human CF bronchial epithelial (CFBE) cell lines expressing a wild-type (WT) or mutant (deltaF508) CF transmembrane conductance regulator (CFTR) channel with human vascular endothelial cells and investigate the effects of bicarbonate. Vascular endothelial cells induced better barrier properties in CFBE cells as reflected by the higher resistance and lower permeability values. Activation of CFTR by cAMP decreased the electrical resistance in WT but not in mutant CFBE cell layers confirming the presence and absence of functional channels, respectively. Sodium bicarbonate (100 mM) was well-tolerated by CFBE cells: it slightly reduced the impedance of WT but not that of the mutant CFBE cells. Sodium bicarbonate significantly decreased the more-alkaline intracellular pH of the mutant CFBE cells, while the barrier properties of the models were only minimally changed. These observations indicate that sodium bicarbonate is beneficial to deltaF508-CFTR expressing CFBE cells. Thus, sodium bicarbonate may have a direct therapeutic effect on the bronchial epithelium.

## 1. Introduction

Despite recent advances, the pharmacological therapy of cystic fibrosis (CF), an autosomal recessive genetic disorder caused by the mutation of the cystic fibrosis transmembrane conductance regulator (CFTR or ABCC7) gene, is still a largely unmet medical need. Mutations related to the CFTR gene can result in the CFTR protein misfolding, decreased trafficking to and stability in the plasma membrane of epithelial cells and an impaired activity of this ATP-gated anion channel [1]. This is the reason why CF is considered a disease of impaired protein folding and function, and rescue of the cells from stress due to chronic expression of misfolded proteins, including mutant CFTR, can improve the loss-of-function phenotype [2]. Used in triple combination, small molecular CFTR modulators, like correctors, which act as pharmacological chaperones or proteostasis regulators, and potentiators, which directly improve CFTR channel functions, now offer a breakthrough therapy for the most common CFTR mutations [3,4]. Patients carrying the most common ΔF508-CFTR mutations can benefit from these new drugs, however these therapies are either not suitable for all patients, who might be unresponsive due to rare or unique CFTR mutations, or they cannot afford them due to their excessive cost [5].

CF is a multi-organ disease with progressive decline in lung function, therefore the care of CF patients requires a multidisciplinary and multifaceted approach including pharmaco- and physiotherapy as well as psychosocial interventions [6]. Adjuvant therapies also play an important role in the management of CF, especially to prevent the accumulation of viscous mucus and bacterial infection in the respiratory tract. CFTR and bicarbonate secretion are central elements in the regulation of mucus viscosity. CFTR not only functions as a cAMP/PKA-regulated epithelial anion channel, but also controls the activity of other ion channels and transporters, such as the epithelial Na^+^ channel (ENaC) or the Cl^-^/HCO_3_^-^ exchanger SLC26A4 [7]. Gene mutations or exogenous noxae (i.e., cigarette smoke) can impair CFTR functions compromising transepithelial electrolyte and water transport in the respiratory tract [8]. In recent years, a large number of evidence suggests that defective HCO_3_^-^ secretion plays a central role in the pathogenesis of CF- and non-CF-related airway diseases [7,8]. Importantly, HCO_3_^-^ controls the luminal pH in the airways. It has been demonstrated in a porcine model of CF that the acidic pH in the trachea reduced the bacterial-killing capacity of antimicrobial peptides, such as lysozyme and lactoferrin, which was corrected by administration of nebulized sodium bicarbonate [9]. More recently, it has been reported that the antibacterial effects of defensins and LL-37 peptide are largely pH-dependent [10]. Sodium bicarbonate not only restores the activity of antimicrobial peptides by regulating pH in the airway surface liquid, but has a direct bacteriostatic effects and inhibits biofilm formation of CF-related bacteria [11]. It is also noteworthy, that HCO_3_^-^ is necessary for chelating protons and Ca^2+^ for proper unfolding of secreted mucin molecules which helps to maintain the normal viscosity of the airway surface liquid [12].

To better understand CF and develop new treatments, relevant model systems are needed. Immortalized airway epithelial cells have been proven to be particularly useful experimental tools [13]. Several cell lines exist to study CF, especially CFTR mutations, the respective phenotypes and pharmacological therapies. Although in some studies non-respiratory cell lines (e.g., HeLa) transfected with wild-type (WT) or mutant CFTR are used to investigate the function of the CFTR channel, differences were found in both receptor trafficking and activation as compared to transfected respiratory cell lines [14]. The human CF bronchial epithelial cell line CFBE41o^–^ (CFBE), developed by Kunzelmann et al. [15], forms adherent, tight and polarized cell layers and expresses a transcriptome that is similar to that of primary airway epithelial cells [16]. This cell line, which does not express endogenous CFTR at mRNA or protein level, has been transfected by several groups to generate cell lines expressing wild-type or ΔF508 CFTR [14,16,17]. This human cell line model from a relevant tissue type maintains many features necessary for studies on CFTR function and treatment with small drug molecules at the cellular level. To increase the complexity and similarity of respiratory culture models to the lung tissue, in vitro co-culture models were introduced. Indeed, an increasing correlation was found between airway models and in vivo tissue by the combination of multiple lung-relevant cell types [18]. In these models in addition to alveolar epithelial cells, vascular endothelial cells and/or immune cells, like macrophages or mast cells are used [18]. However, few co-culture models exist for bronchial epithelium. Recently a co-culture model of human bronchial epithelial cells with human microvascular endothelial cells was described and optimized for microscopical studies [19]. However, no such co-culture models have been established for the study of CF.

Experimental and clinical results with inhaled sodium bicarbonate as an adjuvant therapy in CF are promising due to its mucolytic and bacteriostatic properties, but its direct effect has yet not been studied on respiratory epithelial cells. Our aim was to establish and characterize co-culture models of human CF bronchial epithelial cell lines expressing wild-type or ΔF508-CFTR channels with human vascular endothelial cells and investigate the effects of bicarbonate. Specifically, our study focused on the sodium-bicarbonate-treatment-induced changes in cell impedance, viability, morphology, barrier function, intracellular resting pH and cellular localization of the CFTR channel.

## 2. Results

### 2.1. Characterization of the Barrier Properties of the CFBE Cells in Monoculture and in Co-Culture with Endothelial Cells

**Figure 1:**
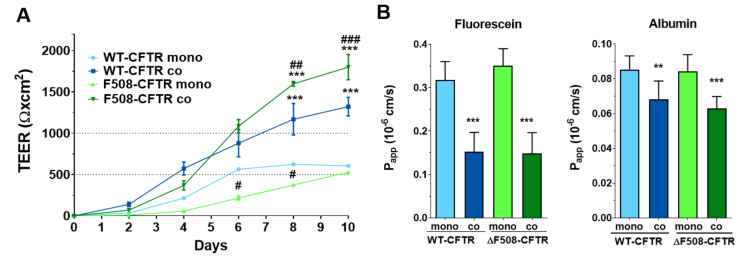
Transepithelial electrical resistance (TEER) (**A**) and permeability values (apical to basal direction) (**B**) of cystic fibrosis bronchial epithelial (CFBE) monocultures or co-cultures with endothelial cells. Values are presented as means ± SD, *n* = 4/group. Statistical analysis: 2-way ANOVA and Bonferroni test. ** *p* < 0.01, *** *p* < 0.001 compared to the monocultures; ^#^ *p* < 0.05, ^##^ *p* < 0.01, ^###^ *p* < 0.001 compared to the respective wild-type group.

Both CFBE cell lines formed good barriers by day 10 on culture inserts, the transepithelial electrical resistance (TEER) values were above 500 Ω × cm^2^ (Figure 1A) and the P_app_ for both permeability markers were low, in the range of 10^−7^ cm/s (Figure 1B). The integrity of bronchial epithelial layers was increased more than 2- and 3-fold in the presence of human vascular endothelial cells as reflected by the higher resistance values (WT-CFTR CFBE cells monoculture: 605 ± 7, co-culture: 1322 ± 112 Ω × cm^2^; ΔF508-CFTR CFBE monoculture: 529 ± 16, co-culture: 1800 ± 152 Ω × cm^2^). Endothelial cells also induced lower permeability values (Figure 1B) for fluorescein (WT-CFTR CFBE monoculture: 0.32 ± 0.04, co-culture: 0.15 ± 0.04 10^−6^ cm/s; ΔF508-CFTR CFBE monoculture: 0.35 ± 0.03, co-culture: 0.15 ± 0.05 10^−6^ cm/s) and albumin (WT-CFTR CFBE monoculture: 0.08 ± 0.01, co-culture: 0.06 ± 0.01 10^−6^ cm/s; ΔF508-CFTR CFBE monoculture: 0.08 ± 0.01, co-culture: 0.06 ± 0.01 10^−6^ cm/s).

**Figure 2:**
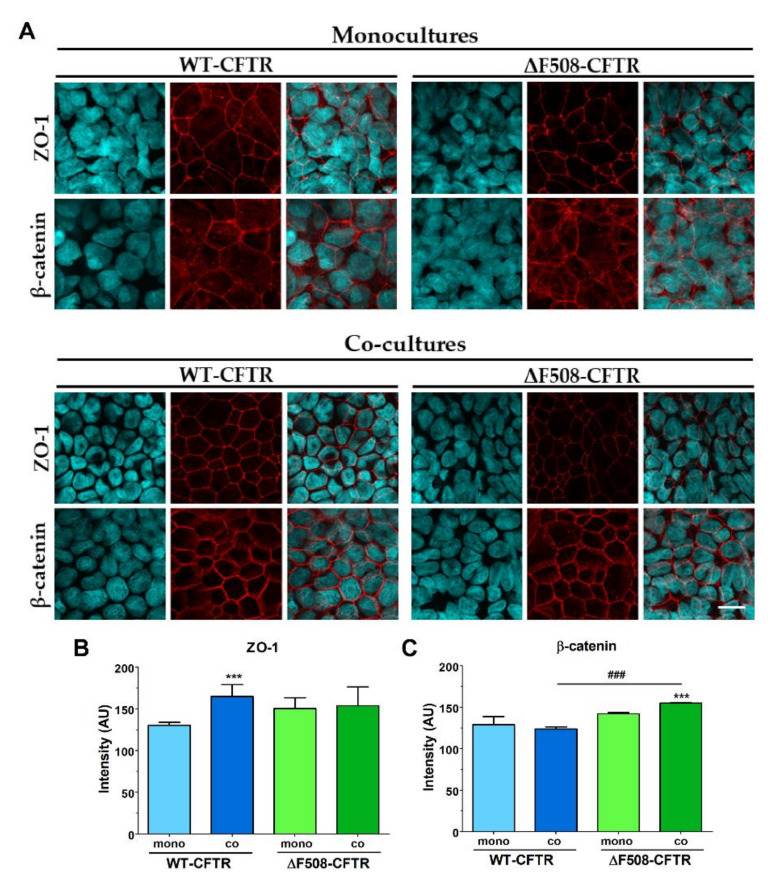
Immunostaining for junctional proteins zonula occludens-1 (ZO-1) and β-catenin after 10 days of monoculture or co-culture with endothelial cells (**A**). The mean pixel intensity of ZO-1 (**B**) and β-catenin (**C**) staining at the cell border. Values are presented as means ± SD, *n* = 3–6/group. Statistical analysis: 2-way ANOVA and Bonferroni test. *** *p* < 0.001 compared to the monocultures. ^###^ *p* < 0.001 compared to WT-CFBE cells. Red color: immunostaining for junctional proteins. Cyan color: staining of cell nuclei. Bar: 25 µm.

To evaluate junctional morphology, the tight-junction-associated cytoplasmic linker zonula occludens protein-1 (ZO-1), the adherens junction integral membrane protein E-cadherin and its linker protein, β-catenin, were selected. The co-culture conditions also increased the tightness of the interepithelial junctions and made epithelial cells to form a better monolayer visualized by immunostaining for ZO-1 and β-catenin junctional proteins (Figure 2A). The mean pixel intensity of ZO-1 staining at the cell border was higher in the case of the WT-CFTR CFBE cells (Figure 2B), while stronger β-catenin intensity was observed in the ΔF508-CFTR CFBE cells (Figure 2C). The localization of the immunosignal was stronger and sharper at the cell border in the junctional area of CFBE cell lines when they were grown together with endothelial cells (Figure 2A).

**Figure 3:**
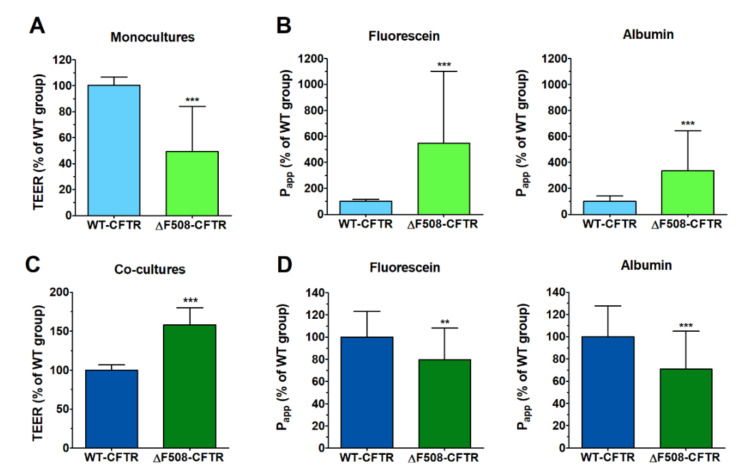
Transepithelial electrical resistance (TEER) (**A**,**C**) and permeability values (**B**,**D**) of CFBE monocultures or co-cultures measured in 8 independent experiments. The values presented as a percentage of the WT-CFTR CFBE group. Values are presented as means ± SD, *n* = 16–52/group. Statistical analysis: 2-way ANOVA and Bonferroni test. ** *p* < 0.01, *** *p* < 0.001 compared to the WT-CFTR CFBE cells.

To compare the barrier integrity of the wild-type and mutant CFBE cells we pooled and analyzed the results of eight independent experiments (Figure 3). Since we found considerable variability in the basal TEER and permeability values of the CFBE cell lines, the values are given as a percentage of the WT-CFTR CFBE groups. Monocultures of the ΔF508-CFTR CFBE cells showed weaker barrier properties as reflected by the lower TEER values (Figure 1 and Figure 3A) and higher permeability values (Figure 3B) for marker molecules compared to the wild-type cells. In contrast, co-culture of ΔF508-CFTR CFBE cells with human vascular endothelial cells resulted in tighter barrier properties as demonstrated by the increased resistance (Figure 1A and Figure 3C), the decreased permeability for the hydrophilic small marker fluorescein and large marker albumin (Figure 3D) and stronger β-catenin staining intensity at the junctional area (Figure 2C).

The culture of CFBE cells at air-liquid interface (ALI), considered as a physiologically more relevant condition, did not result in better barrier properties. As compared to the CFBE cells cultured in a standard way (liquid-liquid interface, LLI) the electrical resistance was lower and more fluorescently labeled molecules went across the cell layers kept in the ALI (Figure S1). Immunostaining of the junctional proteins ZO-1 and E-cadherin also confirm the decreased cell-layer integrity (Figure S2). These results are in accordance with literature data: lower TEER values and an altered staining pattern of junctional proteins were obtained at air-liquid-cultured cell layers due to desiccation and a high rate of apoptosis [17]. Based on these results, standard culture conditions were chosen for the experiments. 

### 2.2. The Effect of CFTR Activator and Inhibitor on the Resistance and Permeability of CFBE Cell Lines

**Figure 4:**
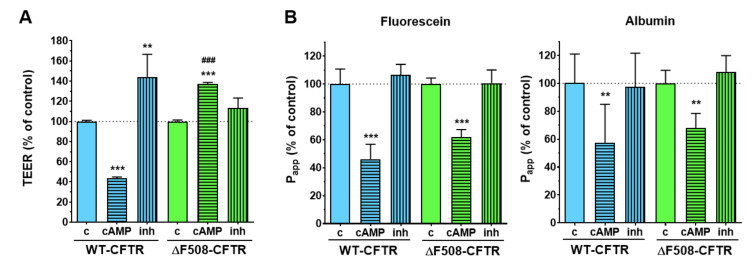
Effects of a cell-permeable cAMP analog (250 µM) and the CFTR channel inhibitor CFTR_inh_-172 (inh; 10 µM) treatment (1 h) on the electrical resistance (**A**) and permeability (**B**) values. Values: percentage of control groups. Means ± SD, *n* = 4/group. 2-way ANOVA and Bonferroni test. ** *p* < 0.01, *** *p* < 0.001 compared to the control groups, ^###^ *p* < 0.001 compared to the respective wild-type group.

Transepithelial electrical resistance (TEER) measures ion movement across cell layers. Activation of the CFTR anion channel by a cell-permeable cAMP analog decreased the electrical resistance to less than half in wild-type, but not in mutant CFBE cell layers (Figure 4A). The permeability values of the wild-type cells for fluorescein and albumin did not increase (Figure 4B). In contrast, less tracer molecules could penetrate across the cell layers, which indicates that the decreased TEER values of the wild-type CFBE cells mean increased ion transport through the activated CFTR channel and not a weaker barrier integrity. ΔF508-CFTR CFBE cells showed increased TEER values and decreased permeability values which reflects a tighter cell layer (Figure 4).

The well-known barrier-tightening effects of cAMP were observed in both cell lines based on the decreased permeability for paracellular tracers (Figure 4B). Our results on ion permeability in the cell line pair are in accordance with previous findings on bronchial epithelial cells demonstrating that cAMP treatment decreased the TEER values of CFBE cells expressing WT-CFTR channels, but increased the resistance of cell layers expressing ΔF508-CFTR channels [20]. Furthermore, the ΔF508-CFTR channels in the CFBE cells, even when expressed at the cell membrane, cannot be stimulated by forskolin-elevating cAMP levels [14].

The 1 h CFTR_inh_-172 treatment had an opposite effect to cAMP: it increased the TEER values of the wild-type CFBE cells (1.4 times higher) while no significant change could be observed in the case of the mutant CFTR-expressing CFBE cells (Figure 4A) indicating that only the WT-CFTR CFBE cells express active CFTR channels. The CFTR-inhibitor treatment did not change the cell-layer integrity for marker molecules (Figure 4B).

### 2.3. The Effect of Cytokines on the Barrier Integrity of CFBE Cell Lines

**Figure 5:**
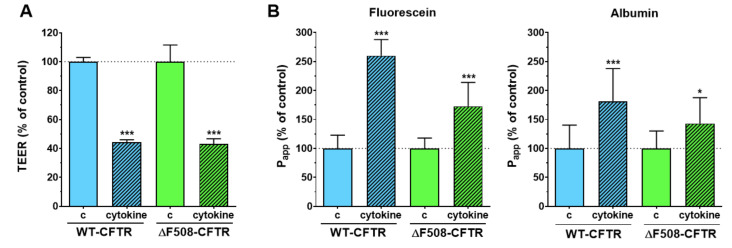
Transepithelial electrical resistance (TEER) (**A**) and permeability values (**B**) of co-cultures after a 6-h cytokine treatment. Values are presented as a percentage of control groups. Means ± SD, *n* = 4/group. Statistical analysis: 2-way ANOVA and Bonferroni test. * *p* < 0.05, *** *p* < 0.001 compared to the control groups.

To mimic inflammatory conditions, CFBE cells were treated with a combination of TNFα (50 ng/mL) and IL-1β (25 ng/mL), as in our previous study on intestinal epithelial cells [21]. The integrity of the cell layers was damaged in both the CFBE cell lines in co-culture conditions after the 6 h cytokine treatment: the resistance decreased to less-than-half of the TEER values of the control groups (Figure 5A). In parallel, the permeability values increased for both markers (Figure 5B) in both cell lines (fluorescein P_app_ in WT-CFTR cells 260%, in ΔF508-CFTR cells 172%; albumin P_app_ in WT-CFTR cells 181%, in ΔF508-CFTR cells 143%). The effect of the cytokines was more robust in the case of the co-cultures (Figure 5) compared to the monocultures, in which a minimal disturbance of the barrier function was observed (Figure S3). These results suggest that co-culture models of CFBE cells are more reactive to cytokines and can be more suitable models to investigate inflammatory conditions in vitro.

### 2.4. The Effect of PN159/KLAL Peptide on the Resistance and Permeability of CFBE Cell Lines

**Figure 6:**
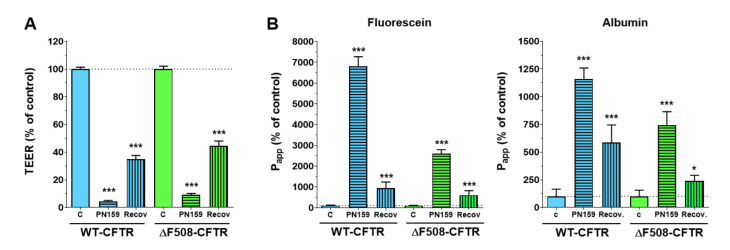
TEER (**A**) and permeability (**B**) values of the CFBE cell layers after a 30-min PN159 peptide treatment and a 24-h recovery period. Values are presented as a percentage of the control groups. Means ± SD, *n* = 4/group. Statistical analysis: 2-way ANOVA and Bonferroni test. * *p* < 0.05, *** *p* < 0.001 compared to the control groups.

To further investigate the response of the CFBE cell line pair we tested a tight-junction modulator and cell-penetrating peptide, PN159/KLAL, that has been well-characterized in our previous studies [22,23]. After 30 min of peptide treatment the TEER values of CFBE cells dropped to below 10% of the level of the control groups (Figure 6A). At the same time, the permeability of the two tracer molecules increased by more than 2 or 3 orders of magnitude (WT-CFTR cells: 6801% of control SF and 1195% of control EBA, ΔF508-CFTR cells: 2606% of control SF and 742% of control EBA) indicating a full opening of the barrier (Figure 6B). After a 24-h recovery period, the TEER values increased but did not reach the values of the control groups (WT-CFTR cells: 35% of control, ΔF508-CFTR cells: 45% of control) (Figure 6A). When the peptide was removed, the permeability values also returned close to the baseline level, although they remained higher.

**Figure 7:**
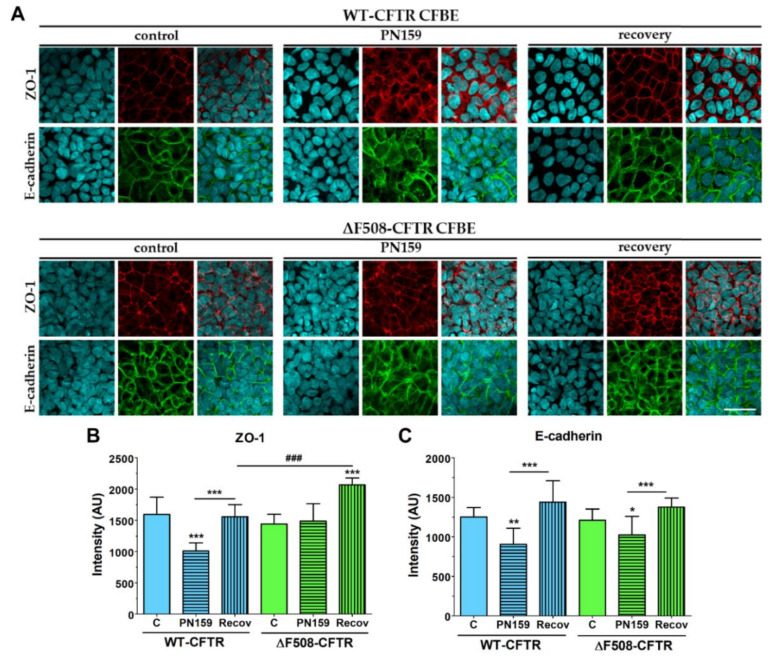
Immunostaining for junctional proteins ZO-1 and E-cadherin CFBE cells without or after a 30-min PN159 peptide treatment, and following a 24-h recovery period (**A**). The mean pixel intensity of ZO-1 (**B**) and E-cadherin (**C**) staining. Means ± SD, *n* = 3–6/group. 2-way ANOVA & Bonferroni test. * *p* < 0.05, ** *p* < 0.01, *** *p* < 0.001 compared to the control and the treated group. ^###^ *p* < 0.001 compared to wild-type epithelial cells. Green and red color: immunostaining for junctional proteins. Cyan color: staining of cell nuclei. Bar: 40 µm.

The immunostaining of junctional proteins confirmed that the peptide opens the intercellular junctions (Figure 7).

The continuous pericellular belt-like ZO-1 and E-cadherin staining pattern, as seen in the control groups, became weaker and disorganized and a cytoplasmic redistribution of the junctional proteins from cell borders was observed. These observations were also confirmed by the analysis of the images taken on the fluorescent immunostaining of the junctional proteins. Peptide treatment resulted in decreased mean pixel intensity of ZO-1 and E-cadherin staining in the junctional area in case of the wild-type cells (Figure 7B,C). The junctional opening effect of the peptide was obvious not only from the functional measurements, but the significantly decreased E-cadherin staining at the cell border of the ΔF508-CFTR cells (Figure 7C). These morphologic changes caused by the peptide were reversible after a 24-h recovery period. Our data indicate that the PN159/KLAL peptide is efficient to open junctions in bronchial epithelial cells; this effect is reversible and the cell-line pair reacted in a similar way to the peptide.

### 2.5. The Effect of Sodium Bicarbonate on Impedance Kinetics and Viability of CFBE Cells

**Figure 8:**
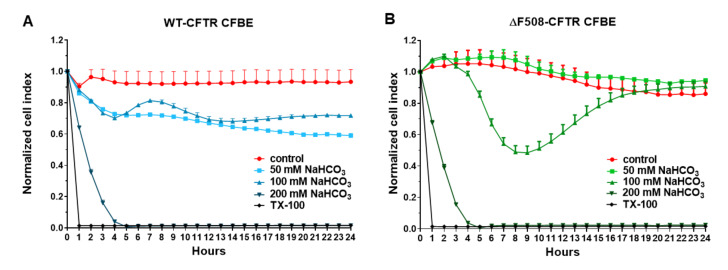
Impedance kinetics measurements of the WT-CFTR CFBE (**A**) and ΔF508-CFTR CFBE (**B**) cells after sodium bicarbonate treatment at different concentrations (50, 100, 200 mM). Culture medium in the control group contained 26 mM sodium bicarbonate. The effects of sodium bicarbonate on the impedance are shown as a normalized cell index. Values are presented as means ± SD, *n* = 11–13.

Sodium bicarbonate showed a concentration- and time-dependent effect on the CFBE cells measured by real-time impedance kinetics (Figure 8 and Supplementary Material Figure S4). The culture medium contained 26 mM NaHCO_3_ in the control group. Sodium bicarbonate at 50 and 100 mM concentrations did not change the cell index of the WT-CFBE cells at the 1-h time point (Figure S4A), but reduced the impedance at later time points (Figure 8A). In contrast to wild-type CFBE cells, the impedance of the cells expressing mutant CFTR was not changed by 50 mM sodium bicarbonate, while treatment with sodium bicarbonate at 100 mM concentration showed a temporary decrease (at 6–15 h) then returned to the level of the control cells in the 16–24-h period (Figure 8B and Figure S4). The highest (200 mM) concentration of sodium bicarbonate caused cell death as reflected by the decrease in impedance of both cell types to the level of the reference compound Triton X-100 detergent.

**Figure 9:**
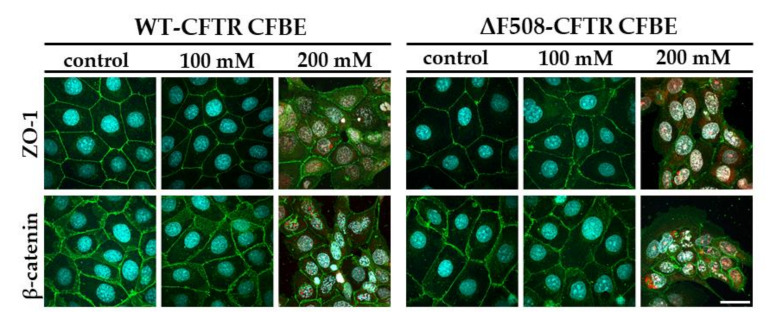
Morphology of CFBE cells after sodium bicarbonate treatment at different concentrations. Cells were treated with culture medium containing 26 (control group), 100 and 200 mM sodium bicarbonate for 24 h. Green color: immunostaining for junctional proteins. Cyan color: cell nuclei. Red color: nuclei of damaged cells. Bar: 40 µm.

The results of the impedance measurements were confirmed by morphological findings (Figure 9). Sodium bicarbonate at 50 and 100 mM concentrations did not change the staining pattern of the junctional proteins as compared to the control group after 24 h of treatment, in agreement with the results of the impedance data shown on Figure 8. Only the 200 mM sodium bicarbonate concentration was toxic to the cells, which resulted in damage to the layer integrity. The plasma membrane of the CFBE cells became permeable to the ethidium homodimer-1 dye which stained the cell nuclei only in the highest sodium bicarbonate treatment group (Figure 9). Based on these results the 100 mM sodium bicarbonate concentration, which has been found to be bacteriostatic in a recent work [11] was selected for the further experiments.

### 2.6. The Effect of Bicarbonate on the Barrier Integrity of CFBE Cells

**Figure 10:**
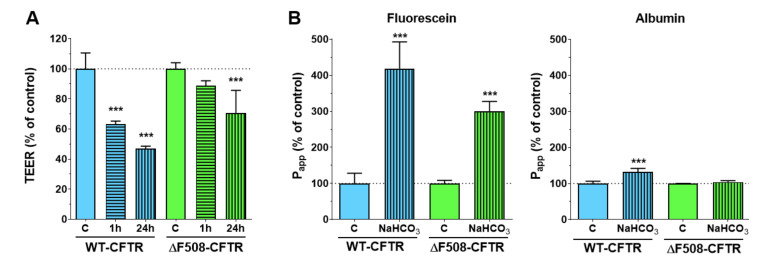
The effect of sodium bicarbonate on the barrier integrity of CFBE cells in co-culture. Transepithelial electrical resistance (TEER) values at 1 and 24 hour time points (**A**) and permeability values (**B**) of the co-culture models after treatment with culture medium containing 26 mM (control group) or 100 mM sodium bicarbonate. Values are presented as a percentage of the control groups. Means ± SD, *n* = 4/group. Statistical analysis: 2-way ANOVA and Bonferroni test. *** *p* < 0.001 compared to the control groups.

To reveal the effect of sodium bicarbonate on barrier integrity, CFBE models in monoculture (Figure S5) and co-culture settings (Figure 10) were treated with culture medium containing 100 mM sodium bicarbonate for 24 h. TEER was also measured after 1 h of treatment (Figure 10A and Figure S5A). CFBE cells expressing wild-type CFTR channels were more sensitive to the bicarbonate treatment, as reflected by the lower TEER values at the two treatment time points (at 1 h: WT-CFTR cells: 57% of control, ΔF508-CFTR cells: 73% of control; 24 h: WT-CFTR cells: 36% of control, ΔF508-CFTR cells: 44% of control). The permeability value for the small hydrophilic marker fluorescein was 4.18 times higher and for the large marker albumin it was 1.3 times higher compared to the control group in co-cultures (Figure 10B). In CFBE cells kept in monoculture the permeability changes for fluorescein were smaller, while the P_app_ for albumin was unchanged (Figure S5B).

**Figure 11:**
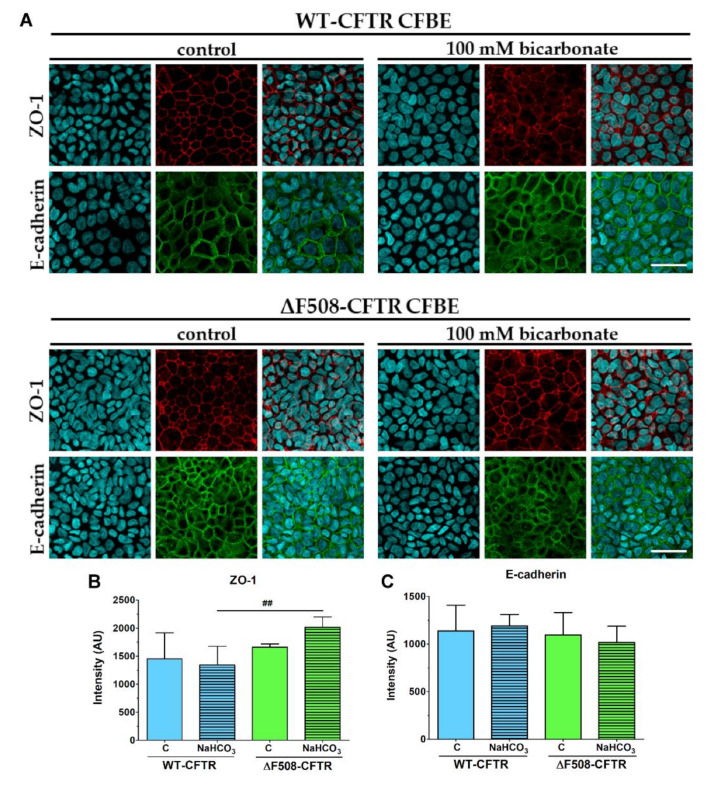
The effect of sodium bicarbonate on the junctional morphology of CFBE cells. Immunostaining of the co-culture model for junction proteins zonula occludens-1 (ZO-1) and E-cadherin (**A**) after treatment with culture medium containing 26 mM (control group) or 100 mM bicarbonate treatment (24 h). The mean pixel intensity of ZO-1 (**B**) and E-cadherin (**C**) immunostainings at the junctional area. Values are presented as means ± SD, *n* = 3–6/group. Statistical analysis: 2-way ANOVA and Bonferroni test. ^##^ *p* < 0.01 compared to the wild-type epithelial cells. Red color: immunostaining for ZO-1. Green color: immunostaining for E-cadherin. Cyan color: cell nuclei. Bar: 40 µm.

The 24-h treatment with culture medium containing 100 mM sodium bicarbonate did not visibly alter the staining pattern for junctional proteins E-cadherin and ZO-1 in CFBE cells in co-culture as compared to the control group (Figure 11). The analysis of junctional staining intensity also confirmed these results (Figure 11B,C), moreover a beneficial effect was observed in the case of the CFBE cells expressing the mutant CFTR channel: the sodium-bicarbonate treatment enhanced the mean pixel intensity of the ZO-1 protein immunostaining at the cell border (Figure 11B).

In agreement with data that cells expressing both types of CFTR channels tolerated well the elevated bicarbonate concentration in their culture medium (Figure 8, Figure 9 and Figure 10) and that the barrier integrity changed only for small ions and tracer molecules of passive diffusion (Figure 10B) in all groups, a preserved, belt-like pericellular staining was visible for both junctional proteins (Figure 11). The same observations were made in the case of monocultures of the CFBE cell lines (Figure S6).

### 2.7. The Effect of Bicarbonate on the Resting Intracellular pH of CFBE Cells

**Figure 12:**
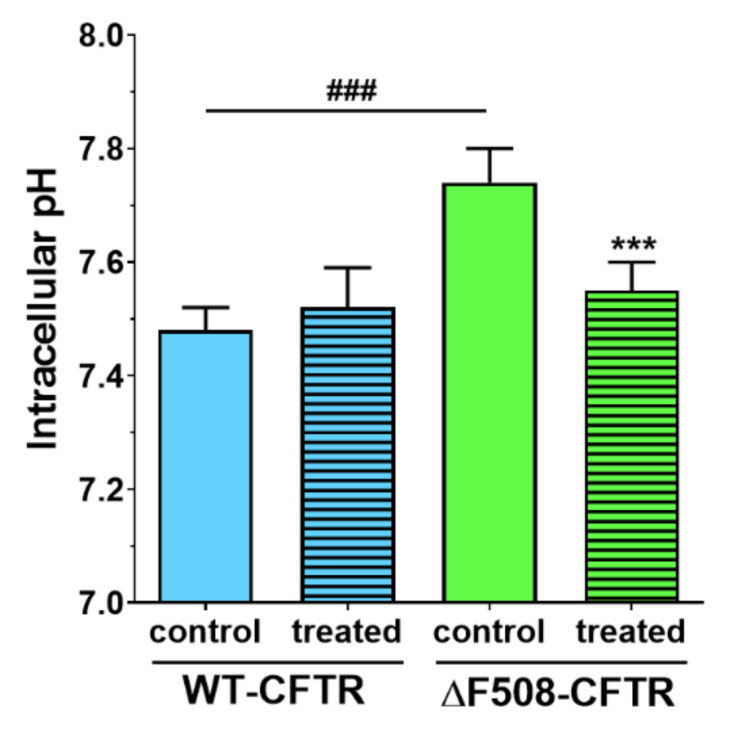
The effect of sodium bicarbonate treatment on the resting intracellular pH of CFBE cells. The pH_i_ of WT-CFTR and ΔF508-CFTR CFBE cells after a 1-h treatment with buffer containing 26 mM (control group) or 100 mM sodium bicarbonate. Values are presented as means ± SD, *n* = 47–67. Statistical analysis: 2-way ANOVA followed by Bonferroni test. ^###^ *p* < 0.001 compared to the wild-type epithelial cells, *** *p* < 0.001 compared to the control groups.

The resting intracellular pH (pH_i_) of the untreated WT-CFTR CFBE cells was found to be 7.48 ± 0.04 which was not changed by treatment with 100 mM NaHCO_3_. The cells expressing the mutant CFTR channel showed significantly elevated pH_i_ (7.74 ± 0.06) as compared to the WT cells (Figure 12). This finding is in accordance with a previous observation of Walker et al., who found that the intracellular pH of CFTR-KO crypt epithelial cells is more alkaline when compared to the wild-type cells [24]. Importantly, we found that NaHCO_3_ treatment of ΔF508-CFTR CFBE cells decreased the pH_i_ to the level of the pH_i_ measured in WT cells, indicating a beneficial effect on the mutant cells.

### 2.8. The Effect of Bicarbonate on the Immunolocalization of CFTR Channel in CFBE Cells

**Figure 13:**
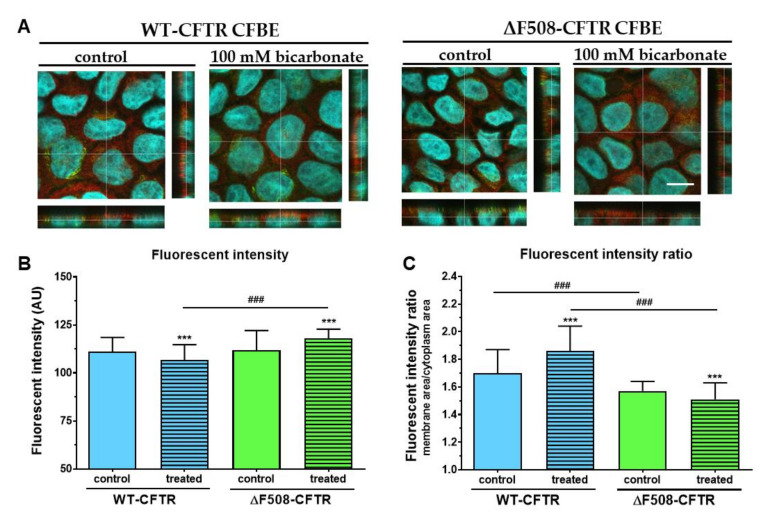
The effect of sodium bicarbonate on the immunolocalization of CFTR channels in CFBE cells. (**A**) WT-CFTR and ΔF508-CFTR CFBE cells were treated with culture medium containing 26 mM (control group) or 100 mM sodium bicarbonate for 24 h. Red color: immunostaining for CFTR. Green color: WGA lectin staining. Cyan color: cell nuclei. Bar: 10 µm. Image analysis of the immunostainings showing (**B**) mean pixel intensities of the CFTR staining in the membrane region of the treatment groups and (**C**) the ratio of mean pixel intensities of the CFTR staining in the membrane region divided by mean pixel intensities of the CFTR staining in the cytoplasm regions. Values are presented as means ± SD, *n* = 4018 image stacks/group. Statistical analysis: 2-way ANOVA and Bonferroni test. ^###^ *p* < 0.001 compared to the wild-type epithelial cells, *** *p* < 0.001 compared to the control groups.

Both cell lines gave immunosignals for CFTR using a polyclonal antibody (Figure 13A). To determine the apical membrane region of the cells, wheat germ agglutinin (WGA) lectin staining was performed which labeled the plasma membrane glycocalyx. The immunostaining pattern in the confocal microscopy images looked diffuse and in the z-direction the fluorescent signal was observed in both the apical and cytoplasmic regions. Using image analysis, the mean pixel intensities of the CFTR immunostaining in apical regions close to the plasma membrane and the remaining cell (cytoplasm) regions were determined. The mean pixel intensities in apical regions of the CFBE cell-line pair were very close (WT-CFTR CFBE cells: 111.09 ± 7.35 vs. ΔF508-CFTR CFBE cells: 111.86 ± 10.17) (Figure 13B). Treatment of CFBE cells with culture medium containing sodium bicarbonate at 100 mM concentration decreased the mean pixel intensity of CFTR immunostaining in the apical regions of WT-CFTR CFBE cells (106.73 ± 8.00) but increased it in ΔF508-CFTR CFBE cells (117.85 ± 4.91). The ratios of the CFTR immunostaining pixel intensities in the apical and cytoplasmic regions were significantly higher in the CFBE cells expressing wild-type channels (Figure 13C) than in cells expressing the mutant channels (1.70 ± 0.17 vs. 1.57 ± 0.07) indicating more channels in the apical (membrane) region in WT-CFTR CFBE cells, in concordance with literature data [14].

Sodium bicarbonate treatment further increased the CFTR immunofluorescence intensity ratio in WT-CFTR CFBE cells (1.86 ± 0.18 vs. 1.70 ± 0.17 in the respective control group), while an opposite effect was measured in ΔF508-CFTR CFBE cells (1.51 ± 0.12 vs. 1.57 ± 0.07 in the respective control group). During the morphological analysis we observed that the average height of the WT-CFTR CFBE cells was lower than that of the mutant cell line (6.4 ± 0.7 µm vs. 8.1 ± 0.5 µm), which might indicate an altered cell-volume regulation.

## 3. Discussion

We have successfully established and characterized a new, physiologically more relevant co-culture model of cystic fibrosis using the CFBE cell line. This cell line, derived from samples from a CF patient, is widely used for studies on disease pathology and to test possible pharmacological treatments [25,26]. To better understand CF pathology, CFBE cells, which do not express endogenous CFTR, were transfected with WT and ΔF508-CFTR channels by several groups [14,17,20]. Bebők et al. have characterized in detail a CFBE cell-line pair sharing the same genetic background but expressing either the WT or the mutant channel [14], and this model was further developed in our experiments. Many studies prove that CFBE cells expressing WT and ΔF508-CFTR channels are a useful model system to study biogenesis, trafficking and regulation of this anion channel. The advantages of CFBE cells include easy culture of the cells, robust expression of the respective CFTR channels, epithelial characteristics, like a polarized phenotype and apical expression of CFTR in wild-type cells, and the expression of adenosine and adrenergic receptors which regulate CFTR activity [14]. We could confirm, that both CFBE cell lines formed tight barriers, showed high TEER values and well-established intercellular junctions in concordance with literature data [20]. We also demonstrated the expression of functional CFTR channels in the WT-CFTR CFBE cells, but not in the ΔF508-CFTR CFBE cells. Intracellular cAMP signaling is linked to CFTR activation and increased ion flux, which was reflected in the WT-CFTR CFBE cells as a decrease in TEER values, while the opposite effect was observed after treatment with a CFTR inhibitor. In contrast, the TEER values of the ΔF508-CFTR CFBE cells increased, which is due to the well-known barrier-tightening effects of cAMP [27] as indicated by the decreased paracellular permeability for tracer molecules of diffusion in both cell lines. These functional data are in accordance with the positive immunostaining for CFTR in the cells in our study, and functional TEER results from the literature [20,28], and indicate the validity of our models.

Co-culture models of airway epithelial cells are increasingly used as a more complex experimental setup [18,26]. However, these models are made either from non-bronchial, mostly alveolar epithelial cells [18] or are used as 3D organoids, which are not suitable for permeability studies [26]. The only study we could find presenting a human bronchial epithelial and vascular endothelial co-culture was a microfluidic model optimized for microscopy [19]. Thus, our study is the first to establish and characterize a culture-insert-based co-culture model using a CFBE cell-line pair and human vascular endothelial cells. We found that the presence of endothelial cells induced better barrier properties in CFBE bronchial epithelial cells. The resistance values increased while the permeability values decreased for marker molecules. The co-culture conditions also increased the tightness of interepithelial junctions and made CFBE cells form a well-organized, tighter monolayer visualized by immunostaining. We observed, that the WT-CFBE model showed lower, while the ΔF508-CFTR CFBE model had significantly higher, TEER.

When the barrier integrity of the CFBE cell-line pair was compared, based on several independent experiments, we confirmed that monocultures of ΔF508-CFTR CFBE cells showed lower TEER and higher paracellular permeability values indicating weaker barrier properties. These findings are in agreement with literature data, in which bronchial epithelial cell monolayers expressing ΔF508-CFTR have a higher paracellular permeability [26,28]. These papers not only link CFTR dysfunction to the dysregulation of the paracellular transport route [28], but also demonstrate that the expression of the WT-CFTR restores tight-junction localization and function in cystic fibrosis bronchial epithelial cells [26]. Our novel finding is, that when ΔF508-CFTR CFBE cells are kept in co-culture with human vascular endothelial cells from a normal genetic background, this effect is rescued: the mutant cells show tighter barrier properties: increased resistance and decreased permeability for hydrophilic marker molecules.

Although air-liquid interface culture conditions are generally considered as better reflecting physiological conditions [26], in our hands culturing CFBE cells in air-liquid interface showed weaker barrier properties compared to the traditional liquid submerged cultures. Our results are supported by a study, in which impaired barrier properties were also obtained at air-liquid-cultured CFBE cells [17], indicating that this approach cannot necessarily be applied for all respiratory culture models.

Inflammatory changes in the respiratory system are a hallmark of CF and have been investigated in culture models, where loss of barrier integrity was described in airway epithelial cultures treated with proinflammatory cytokines [26,29]. To test the reaction of the model to inflammatory challenge, co-cultures of CFBE epithelial and endothelial cells were treated with a combination of TNFα and IL-1β. This combination of cytokines caused NF-κB translocation and the loss of barrier integrity in our previous studies on other biological barrier models, namely on intestinal epithelial cells [21] and brain endothelial cells [30]. Treatment with proinflammatory cytokines caused barrier damage in both CFBE cell-based co-culture models as demonstrated by the lower TEER and higher permeability values. This effect was not observed in CFBE epithelial monocultures, where the response of the cells to cytokines was modest. The presence of endothelial cells contributed to the more robust response to proinflammatory cytokine treatment in epithelial cells and suggests that co-cultures can be more suitable models to investigate inflammatory conditions in vitro.

To further characterize the CFBE cell lines we tested their response to the PN159/KLAL peptide, which has a dual effect on barrier cells: it opens the intercellular-tight junctions and also acts as a cell-penetrating peptide [23]. The junction-modulator effect of this synthetic cationic peptide was discovered on human bronchial epithelial cell cultures [31], and we have described a similar effect on models of the intestinal epithelium and the blood-brain barrier [22]. We found that the PN159/KLAL peptide efficiently and reversibly opened the junctions in CFBE bronchial epithelial cells and there was no difference between the reactions of the CFBE cell-line pair to the peptide.

By regulating mucus pH and viscosity, inhaled bicarbonate can be also crucial in the therapy of CF [32]. Indeed, sodium bicarbonate decreased high viscosity of the CF bronchial secretion [33] and a similar effect has been observed in a clinical investigation [34]. This new clinical study on CF patients confirmed the safety and tolerability of nebulized sodium bicarbonate (4.2% and 8.4% solutions) and demonstrated an increased pH and decreased viscosity in bronchial mucus [34]. In addition, sodium bicarbonate (100 mM) inhibited bacterial growth and biofilm formation of bacteria important in CF pathology, indicating it can have an additional beneficial effect in CF therapy [11].

Our study aimed to investigate the direct effect of elevated sodium bicarbonate concentrations on bronchial epithelial cell viability, barrier properties and morphology, which have not been studied yet. Using impedance kinetics to monitor the cell-layer response we confirmed that CFBE epithelial cells tolerate well-elevated sodium bicarbonate concentrations. In our experiments 100 mM sodium bicarbonate (a four-times-higher concentration than in the basal culture medium) was the highest, but still a safe concentration based on viability and cell morphology data. Interestingly, we found differences between the reactions of the CFBE cell lines. CFBE cells expressing the wild-type CFTR channel were more sensitive to higher concentrations of sodium bicarbonate. In ΔF508-CFTR bronchial cells expressing the mutant channel the impedance values were the same after treatment with elevated concentrations of bicarbonate as in the control group. In addition, even a barrier-increasing effect was seen in the case of these ΔF508-CFTR epithelial cells based on the image analysis of junctional protein ZO-1. One of our most important findings is that the barrier function of the CFBE epithelial cells is preserved after sodium bicarbonate treatment based on resistance, permeability and morphological results.

We not only demonstrated that higher concentrations of sodium bicarbonate can be safely used in CFBE cells, but we made observations that these might be also beneficial for cells expressing the mutant CFTR channel. We measured in ΔF508-CFTR bronchial epithelial cells a more alkaline-resting pH_i_ than in CFBE cells expressing wild-type CFTR. Similarly, in CFTR-KO crypt epithelial cells more alkaline pH_i_ was found [24]. Treatment of the cells with 100 mM sodium bicarbonate normalized the pH_i_ of mutant cells to the level of the WT-CFTR CFBE cells.

Although regulation of CFTR expression and targeting is not fully understood, transcription factors and miRNAs are known to regulate these processes [35]. Our results show that administration of 100 mM sodium bicarbonate modifies the apical expression of CFTR in both cell lines. Extracellular HCO_3_^-^ acting as a signal molecule, stimulates the activity of soluble adenylyl cyclase increasing intracellular cAMP concentrations, which in turn activates protein kinase A [36]. Protein kinase A could trigger NF-κB, a transcription factor known to mediate up-regulation of the CFTR gene expression in pulmonary epithelial cells [37]. Thus, it is conceivable that higher HCO_3_^-^-permeability of WT-CFTR expressing cells causes up-regulation of adenylyl cyclase activity increasing apical expression of CFTR. Further investigations are needed to explain the reduced CFTR immunofluorescence ratio in ΔF508-CFTR-expressing cells.

Our morphological analysis indicates that mutant CFBE cells have an increased average height suggesting larger cell volume than WT-CFTR-expressing cells. The important role of CFTR in cell-volume regulation has already been demonstrated by other research groups. For instance, it has been shown that cell-volume regulation is defective of small intestinal crypts isolated from CFTR^-/-^ mutant mice [38]. The authors claimed that this was due to impaired CFTR-dependent regulation of volume sensitive K^+^ channels. In another study, CFTR potentiated and accelerated regulatory-volume decrease following hypotonic challenge by an autocrine mechanism involving ATP release and signaling [39]. Consequently, lack of functional CFTR could lead to impaired ATP secretion and depletion of this autocrine, purinergic signaling system under both basal and hypotonic conditions. Furthermore, altered intracellular Cl^-^ concentrations of CF cells may also contribute to defective cell-volume regulation [40].

In conclusion, we characterized a new co-culture model of bronchial epithelial cells and demonstrated that vascular endothelial cells induced better barrier properties in CFBE cells as reflected by the higher resistance and lower permeability values. Activation of CFTR by cAMP decreased the electrical resistance in wild-type but not in mutant CFBE cell layers confirming the presence and absence of functional channels, respectively. Elevated concentrations of sodium bicarbonate were well-tolerated by CFBE cells: it slightly reduced the impedance of wild-type but not that of the mutant CFBE cells. Sodium bicarbonate significantly increased the junctional staining intensity of ZO-1 protein and decreased the more-alkaline intracellular pH of the mutant CFBE cells, while the functional barrier properties of the models were preserved. Our observations on the direct effects of sodium bicarbonate on bronchial epithelial cells confirm the safe therapeutic use of inhaled sodium bicarbonate.

## 4. Materials and Methods

### 4.1. Materials

Unless specified otherwise, all reagents were purchased from Sigma-Aldrich Ltd. (Budapest, Hungary).

### 4.2. Cell Cultures

The human cystic fibrosis bronchial epithelial (CFBE) cell-line pair was kindly provided by Dr. Zsuzsanna Bebők. The cells expressing the wild-type human CFTR (WT-CFTR CFBE) and the CFTR with the most common ΔF508 mutation (ΔF508-CFTR CFBE) were generated by lentiviral transformation of the human CFBE41o^-^ cell line, which does not express CFTR [14]. CFBE human bronchial epithelial cells were grown under selective pressure in puromycin-containing medium to express the transgenes and used for the experiments until passage 18. The cells were grown in Minimum Eagle Medium (MEM) (Gibco, Life Technologies, Carlsbad, CA, USA) supplemented with 10% fetal bovine serum (Pan-Biotech GmbH, Aidenbach, Germany), stable glutamine (Glutamax, 2 mM), puromycin (2 µg/mL) and 50 μg/mL gentamycin in a humidified incubator with 5% CO_2_ at 37 °C. All plastic surfaces were coated with 0.05% rat tail collagen in sterile, distilled water before cell seeding in culture dishes and the medium was changed every 2 days.

Bronchial epithelial cells were cultured in the presence of human vascular endothelial cells [41,42] to create the co-culture model. The endothelial culture media (ECM-NG, Sciencell, Carlsbad, CA, USA) was supplemented with 5% FBS, 1% endothelial growth supplement (ECGS, Sciencell, Carlsbad, CA, USA) and 0.5% gentamicin. For the permeability measurements the epithelial cells were cultured on inserts (Transwell, polycarbonate membrane, 0.4 μm pore size, 1.12 cm^2^, Corning Costar Co., MA, USA) placed in 12-well plates as mono- or co-cultures for 10 days. To prepare the co-culture model, endothelial cells (≤ P9) were passaged (8 × 10^4^ cells/cm^2^) to the collagen-type-IV- and fibronectin- (both at 100 μg/mL) coated bottom side of tissue culture inserts and bronchial epithelial cells were seeded (1 × 10^5^ cells/cm^2^) to the upper side of the membranes which were coated with rat tail collagen. CFBE cells were cultured for 10 days in the presence or absence of endothelial cells and were treated when the cell layer had reached steady resistance values. In the liquid-liquid interface (LLI) culture mode the volume of the medium in the upper compartment was 0.5 mL, in the lower compartment 1.5 mL. For culturing the cells at air-liquid interface (ALI) the medium was removed from the confluent bronchial epithelial cell layer after three days, while the endothelial cells in the bottom compartment received 1 mL fresh culture medium.

### 4.3. Treatments

To functionally validate the presence of wild-type and mutant CFTR channels in the respective cell lines an activator and an inhibitor of CFTR channels were tested on the monocultures. The stock solution (25 mM) of the CFTR channel activator 8-(4-chlorophenylthio)-cAMP (cAMP) was prepared in sterile distilled water and it was further diluted in culture medium at 250 µM final concentration for treatments. To avoid the degradation of cAMP RO 20 1724 (17.5 µM), a cAMP-specific phosphodiesterase 4 inhibitor was added to the treatment solution. For the stock solution (100 mM) the CFTR inhibitor 3-[(3-trifluoromethyl) phenyl]-5-[(4-carboxyphenyl) methylene]-2-thioxo-4-thiazolidinone (CFTR_inh_-172) was dissolved in dimethyl sulfoxide (DMSO), further diluted in culture medium and tested at 10 µM concentration also for 1 h. For both reagents, cell-layer integrity was monitored by electrical resistance and permeability measurements after 1 h of treatment.

To model inflammatory conditions, often observed in CF lung, the mono- and co-cultures were treated with a combination of TNF-α (50 ng/mL) and IL-1β (25 ng/mL) in both compartments of the inserts [21]. After the 6-h cytokine treatment, cell-layer integrity was investigated by electrical resistance and permeability measurement.

To test the similarities and differences between the response of the CFBE cell lines to different treatments we also tested the effect of the PN159/KLAL peptide (NH_2_-KLALKLALKALKAALKLA-amide). This peptide, which shows both cell-penetrating effects and opens intercellular junctions, has been characterized in detail by our research group [22,23]. The stock solution of the peptide (5 mM) was prepared in DMSO, the final concentration of the peptide in cell culture medium was 10 µM, as used in our previous studies. We treated the CFBE monoculture with the peptide for 30 min (only in upper compartments, where the apical junctional complex is situated). After the treatment medium was changed, TEER and permeability measurements were performed immediately and at the end of the 24 h recovery period.

To test the effect of sodium bicarbonate we prepared 50, 100 and 200 mM treatment solutions. The MEM culture medium already contains 26 mM sodium bicarbonate. To achieve a 500 mM stock solution we dissolved 200.5 mg NaHCO_3_ in 5 mL cell culture medium, then sterilized with a 0.2 µm syringe filter. The treatment solutions were diluted from the stock solution in culture medium and were freshly prepared before the experiments. For the viability assay 50, 100 and 200 mM concentrations, for the cell-layer integrity and intracellular pH measurements 100 mM concentration of bicarbonate were used (only upper, apical compartments were treated to mimic inhaled sodium bicarbonate treatment).

### 4.4. Measurement of the Electrical Resistance of CFBE Cell Layers

Transepithelial electrical resistance (TEER) indicates the integrity and paracellular permeability of cell layers for ions. TEER reflects the tightness of the intercellular junctions closing the paracellular cleft, therefore the overall tightness of cell layers of biological barriers. Before measuring the transepithelial electrical resistance (TEER) of the cell layers 500 µl of fresh medium was added to the upper and lower compartments. The measurement was performed after 15 min of equilibration time in the CO_2_ incubator. We used an EVOM volt-ohmmeter (World Precision Instruments, Sarasota, FL, USA) combined with STX-2 electrodes to determine TEER values which were expressed relative to the surface area of the monolayers as Ω × cm^2^. TEER of cell-free inserts (90–100 Ω × cm^2^) was subtracted from the measured data. The culture medium was changed and TEER was checked every second day. TEER values were measured before and right after the experiments.

### 4.5. Permeability Study on CFBE Cell Culture Model

To determine the tightness of the bronchial epithelial culture model two passive permeability marker molecules, fluorescein (10 μg/mL; Mw: 376 Da) and Evans-blue labeled albumin (167.5 μg/mL Evans-blue dye and 10 mg/mL bovine serum albumin; MW: 67.5 kDa) were tested [22]. The inserts were transferred to 12-well plates containing 1.5 mL Ringer buffer in the acceptor (lower/basal) compartments. In the donor (upper/apical) compartments 0.5 mL buffer was pipetted containing both markers. To avoid the unstirred-water-layer effect, the plates were kept on a horizontal shaker (120 rpm) during the assay. The assay lasted for 30 or 60 min. The concentrations of the marker molecules in the samples from both compartments were determined by a fluorescence multiwell plate reader (Fluostar Optima, BMG Labtechnologies, Germany; fluorescein: 485 nm excitation wavelength, 520 nm emission wavelength; Evans-blue labeled albumin: 584 nm excitation wavelength, 680 nm emission wavelength).

The apparent permeability coefficients (P_app_) were calculated as previously described [23]. Briefly, cleared volume was calculated from the concentration difference of the tracer in the acceptor compartment (Δ[C]_A_) after 30 or 60 min and in donor compartments at 0 h ([C]_D_), the volume of the acceptor compartment (V_A_; 1.5 mL) and the surface area available for permeability (A; 1.12 cm^2^) using this equation:

(1) $$P_{\mathrm{app}}\;(\mathrm{cm}/s)=\frac{\Delta {\left[C\right]}_{A}\;\times \;V_{A}}{A\;\times \;{\left[C\right]}_{D}\;\times \;\Delta t}$$

### 4.6. Cell Viability Measurements

The kinetics of the epithelial cell reaction to the sodium bicarbonate treatment was monitored by impedance measurement at 10 kHz (RTCA-SP instrument, ACEA Biosciences, San Diego, CA, USA). Real-time cell electronic sensing is a non-invasive, label-free, impedance-based technique to quantify the kinetics of proliferation, viability and cellular reaction of adherent cells. This method to follow cell damage and/or protection in living barrier forming cells has been successfully used by our team in the last seven years [23,30]. The 96-well E-plates with built-in gold electrodes were coated with collagen type I (50 μg/mL) and dried for 30 min under sterile airflow. For background measurements 50 μL cell culture medium was added to the wells, then cells were seeded at a density of 1 × 10^4^ cells/well to the coated 96-well plates with integrated gold electrodes (E-plate 96, ACEA Biosciences). Cells were cultured for 3–4 days in a CO_2_ incubator at 37 °C and the impedance of cell layers was monitored every 10 min until the end of experiments by an RTCA-SP instrument (ACEA Biosciences). Cell index was defined as (Rn-Rb)/15, where Rn is the cell-electrode impedance of the well when it contains cells and Rb is the background impedance of the well with the medium alone. Cells were treated with culture medium containing sodium bicarbonate at 50, 100 and 200 mM concentrations at the beginning of the plateau phase of growth and the effects were followed for 24 h. Triton X-100 detergent (1 mg/mL) was used as a reference compound to induce cell death.

### 4.7. Immunohistochemistry

Immunostaining was used to evaluate morphological differences between the two CFBE cell lines when cultured in different conditions (mono- vs. co-culture, LLI vs. ALI) or treated by different concentrations of sodium bicarbonate. Cells were grown on culture inserts and after the treatments they were washed with phosphate buffered saline (PBS) and fixed with 3% paraformaldehyde solution for 15 min at room temperature. The cells were permeabilized by 0.2% TX-100 solution for 10 min and the nonspecific binding sites were blocked with 3% bovine serum albumin in PBS. Primary antibodies rabbit anti-ZO-1 (AB_138452, 1:400; Life Technologies, Carlsbad, CA, USA), rabbit anti-β-catenin (AB_476831, 1:400) and mouse anti-E-cadherin (AB_397580, 1:400; Life Technologies, Carlsbad, CA, USA) were applied as an overnight treatment. Incubation with secondary antibodies Alexa Fluor-488-labeled anti-mouse (AB_2534088, 1:400; Life Technologies, Invitrogen, USA) and anti-rabbit IgG conjugated with Cy3 (AB_258792, 1:400) lasted for 1 h. Hoechst dye 33342 was used to stain cell nuclei. After mounting the samples (Fluoromount-G; Southern Biotech, Birmingham, USA) staining was visualized by confocal laser scanning microscope (Olympus Fluoview FV1000, Olympus Life Science Europa GmbH, Hamburg, Germany).

We used double nuclei staining to evaluate cell-membrane damage. The blue Hoechst dye stains every cell nuclei and the red ethidium homodimer-1 dye stains only the nuclei of damaged cells [23]. CFBE cells were cultured on collagen-coated glass coverslips (Menzel-Glaser, Braunschweig, Germany) in a 12-well plate. Cells were seeded at a 10^5^ cells/well density and cultured for 3 days. Cell layers after reaching confluency were treated with culture medium containing 50, 100 and 200 mM bicarbonate for 24 h. Ethidium homodimer-1 dye (1 μM) was added to the cells in the last 30 min of the 24 h bicarbonate treatment. After a washing step with PBS the cells were fixed with aceton:methanol (1:1) solution for 3 min. Then the samples were processed for immunostaining for junctional proteins ZO-1 and β-catenin as described above.

The localization of CFTR channels in the CFBE cells was studied by concomitant staining of the samples with a rabbit polyclonal anti-CFTR antibody (Alomone Labs, Jerusalem, Israel; 1:400) and wheat germ agglutinin lectin (WGA) conjugated with Alexa fluor 488 (Invitrogen, W11261) which binds to the *n*-acetyl-d-glucosamine and sialic acid residues of the glycocalyx covering the cells’ surface. The cells were fixed with 3% paraformaldehyde, washed with PBS and incubated with WGA (5 µg/mL) in PBS for 10 min at room temperature [43]. This step was followed by permeabilization with 0.2% TX-100 solution for 10 min, blocking with 3% bovine serum albumin in PBS for 1 h and incubation with the CFTR antibody overnight. After the washing steps cells were incubated with secondary antibody Cy3-conjugated anti-rabbit IgG for 1 h. Cell nuclei were stained with Hoechst dye 33342. Samples were mounted and the staining was visualized by a Leica TCS SP5 confocal laser scanning microscope (Leica Microsystems GmbH, Wetzlar, Germany).

The cellular localization of the CFTR channels was determined by image analysis (MATLAB, The Mathworks Inc, Natick, Ma, USA) performed on the immunostained CFBE image stacks. First, the image stacks of the WGA staining were used to determine the apical region of the imaged cells. For this the WGA image stacks were laterally smoothed by a 10-pixel-wide 2D Gaussian filter. Within each column (z-direction) of a smoothed WGA stack the cells’ apical membrane position was determined by finding the pixel position where the intensity first rises above 30% of the maximum value in the column, going from the top of the image stack (apical side). Next, based on the obtained apical contour of the cells, spatial masks were calculated for the cell membrane and cytoplasm. The membrane mask was calculated as a 12-pixels- (2.4 µm) wide stripe starting from the top edge of the cells. The cytoplasm mask was taken as the area between the bottom of the membrane mask and the bottom of the image stack (basal side). Finally, the spatial masks were used to calculate the mean pixel intensities of the CFTR staining in the membrane and cytoplasm regions (Video S1–S4). A total of 9 images were analyzed in each group consisting of 512 image stacks (4018 image stacks/group).

The intensity of junctional stainings (ZO-1, E-cadherin, β-catenin) were evaluated by image analysis (MATLAB, The Mathworks Inc, Natick, Ma, USA) performed on the immunostained images. A K-means clustering-based image segmentation function was used to segment the images into areas corresponding to the cytoplasm and the junctional regions. Based on the segmentation, spatial masks were obtained and used for calculating the mean pixel intensity in the junctional areas.

### 4.8. Measurement of Resting Intracellular pH

CFBE cells were cultured on rat tail collagen-coated glass coverslips (24 mm) in 35 mm cell culture dishes (Orange Scientific, Braine-l’Alleud, Belgium). The cells were seeded at 2 × 10^5^ cells/dish density and cultured until confluency. Cells treated with 100 mM NaHCO_3_ in cell culture medium were kept for 1 h in a CO_2_ incubator at 37 °C. The cells were washed once with PBS and were loaded with the pH-sensitive fluorescent dye 2’,7’-bis-(2-carboxyethyl)-5-(and-6-) carboxyfluorescein-acetoxymethyl ester (BCECF-AM; Biotium Inc., Fremont, CA, USA) at 2 µM concentration in HEPES-buffered solution (HEPES-BS; 140 mM NaCl, 5 mM KCl, 1 mM CaCl_2_, 1 mM MgCl_2_, 10 mM D-glucose and 10 mM HEPES acid; pH = 7.4) at 37 °C in a wet chamber for 20–30 min. Thereafter, the cells on coverslips were continuously perfused at a rate of 1–1.5 mL/minute in a perfusion chamber (QE-1, Warner Instruments, Hamden, CT, USA) mounted on a Zeiss Axio Observer 7 microscope (Carl Zeiss Microscopy GmbH, Jena, Germany). The cells were excited with light at wavelengths of 495 (±3) nm and 436 (±10) nm, and the 495/436 fluorescence emission ratio was measured at 540 ± 20 nm to determine intracellular pH (pH_i_). Images from fourteen to seventeen randomly selected cells (regions of interests, ROIs) were obtained by using the ZEN software (Carl Zeiss Microscopy GmbH). One measurement was recorded every two seconds. The 495/436 emission ratio of cells perfused with HEPES-BS was determined at the beginning of the measurements. To convert the emission ratio to resting pH_i_, cells were perfused with a high K^+^ HEPES-BS (K-HEPES-BS; 15 mM NaCl, 130 mM KCl, 1 mM CaCl_2_, 1 mM MgCl_2_, 10 mM d-glucose, 10 mM HEPES acid; pH set between 6.0 and 8.4, and 10 µM nigericin sodium salt (Tocris Bioscience, Bristol, UK)) calibration solutions with known pH values [44].

### 4.9. Statistical Analysis

All data presented are means ± SD. The values were compared using the analysis of variance followed by Dunett’s test or two-way ANOVA and Bonferroni post-hoc test posttest using GraphPad Prism 5.0 software (GraphPad Software Inc., San Diego, CA, USA). Changes were considered statistically significant at *p* < 0.05.

## Supplementary Materials

Supplementary Materials can be found at https://www.mdpi.com/1422-0067/21/11/4024/s1. Figure S1. Transepithelial electrical resistance (A) and permeability values (B) of the CFBE co-cultures grown at liquid-liquid interface (LLI) and air-liquid interface (ALI). Means ± SD, *n* = 4/group. Statistical analysis: 2-way ANOVA and Bonferroni test. *** *p* < 0.001 compared to the LLI groups. Figure S2. Immunostaining of junctional proteins ZO-1 and E-cadherin in CFBE cells cultured at liquid-liquid interface (LLI) and air- liquid interface (ALI). Green and red color: immunostaining for junctional proteins. Cyan color: staining of cell nuclei. Bar: 40 µm. Figure S3. Transepithelial electrical resistance (A) and permeability values (B) of monocultures after 6-hour cytokine treatment. Values are presented in the percentage of control groups. Means ± SD, *n* = 4/group. Statistical analysis: 2-way ANOVA followed by Bonferroni test. ** *p* < 0.01, *** *p* < 0.001 compared to the control groups. Figure S4. Impedance measurements of WT-CFTR CFBE (A) and ΔF508-CFTR CFBE (B) cells after treatment with culture medium containing different concentrations of sodium bicarbonate (50, 100, 200 mM). Culture medium in the control groups contains 26 mM sodium bicarbonate. The effects of bicarbonate on the impedance were shown as normalized cell index. Values are presented as means ± SD, *n* = 11–13. Statistical analysis: ANOVA and Bonferroni test. *** *p* < 0.001 compared to the control groups. Figure S5. The effect of sodium bicarbonate on the barrier integrity of CFBE cells in monoculture. Transepithelial electrical resistance values at 1 and 24- hour time points (A) and permeability values (B) of the monoculture models after treatment with culture medium containing 100 mM sodium bicarbonate. Values are presented in the percentage of control groups. Means ± SD, *n* = 4/group. Statistical analysis: 2-way ANOVA followed by Bonferroni test. * *p* < 0.05, ** *p* < 0.01, *** *p* < 0.001 compared to the control groups. Figure S6. The effect of sodium bicarbonate on the junctional morphology of CFBE cells in monoculture. Immunostaining of the monoculture models for junction proteins zonula occludens-1 (ZO-1) and E-cadherin after treatment with culture medium containing 26 mM (control group) or 100 mM bicarbonate treatment (24 h). Red color: immunostaining for ZO-1. Green color: immunostaining for E-cadherin. Cyan color: cell nuclei. Bar: 40 µm.
