# Supplementary material for: The Effect of Sodium Bicarbonate, a Beneficial Adjuvant Molecule in Cystic Fibrosis, on Bronchial Epithelial Cells Expressing a Wild-Type or Mutant CFTR Channel
Source: Int J Mol Sci. 2020 Jun 4;21(11):4024. doi: 10.3390/ijms21114024 (PMC7312297; doi:10.3390/ijms21114024)
Supplement: Supplementary file 1 [file ijms-21-04024-s001.zip › Supplementary/Grof_Supplementary_Materials_submission.docx]

Supplementary Materials

The effect of sodium bicarbonate, a beneficial adjuvant molecule in cystic fibrosis, on bronchial epithelial cells expressing wild type and mutant CFTR channel

Ilona Gróf ^1^, Alexandra Bocsik ^1^, András Harazin ^1^, Ana Raquel Santa-Maria ^1^, Gaszton Vizsnyiczai ^1^, Lóránd Kiss ^2^, Gabriella Fűr ^2^, Zoltán Rakonczay Jr ^2^, Rita Ambrus ^3^, Piroska Szabó-Révész ^3^, Fabien Gosselet ^4^, Pongsiri Jaikumpun ^5^, Hajnalka Szabó ^6^, Ákos Zsembery ^5^, Mária A. Deli ^1*^

**1. Comparison of CFBE cultures kept at liquid-liquid interface and at air-liquid interface**

In addition to standard, liquid-liquid culture conditions the CFBE cell models were also tested at air-liquid interface (ALI), which is considered as a physiologically more relevant condition. However, at least in our hands, the barrier properties of the co-culture models after 6 days of ALI were not better than those of the models kept in medium only (Figure S1). We measured lower transepithelial electrical resistance (Figure S1A) in both WT-CFTR CFBE (27% of the LLI) and ΔF508-CFTR CFBE cells (69% of LLI). The permeability of the WT-CFTR CFBE model kept in ALI was also higher for the small molecule fluorescein and a trend was seen for albumin (Figure S1B). The permeability of the ΔF508-CFTR CFBE model was a similar trend with increased permeability for albumin.


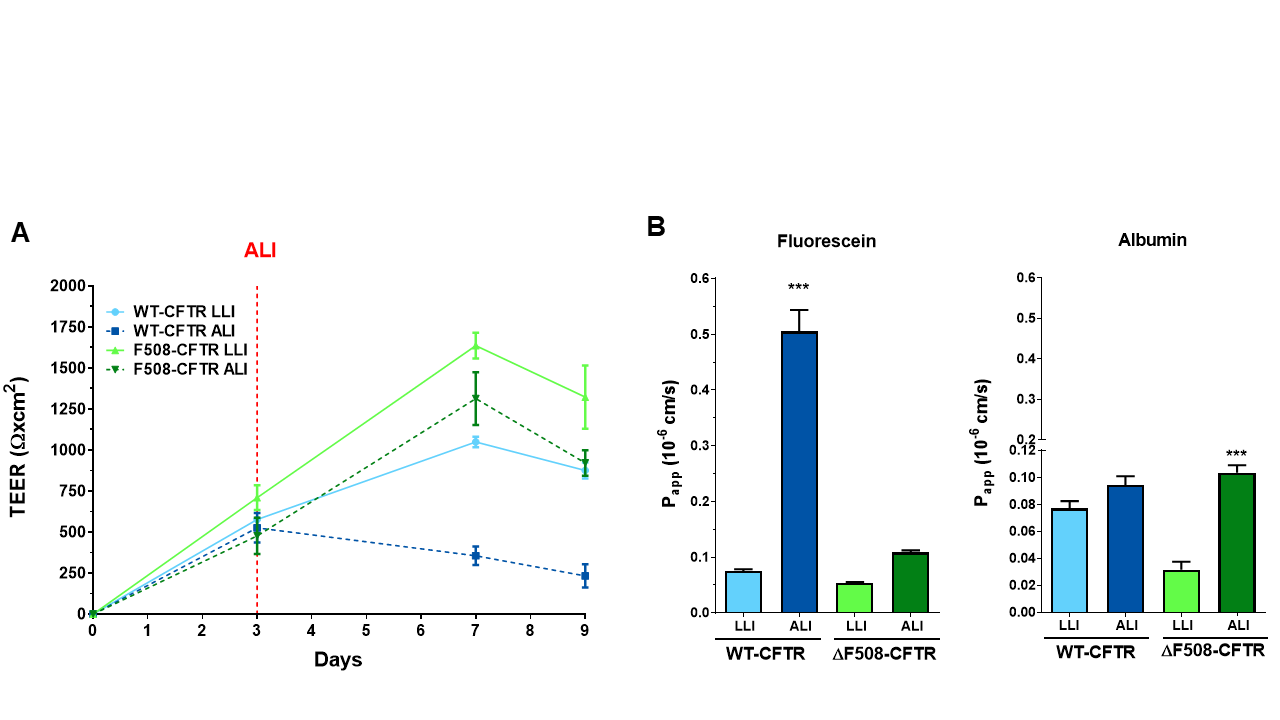


**Figure S1.** Transepithelial electrical resistance (A) and permeability values (B) of the CFBE co-cultures grown at liquid-liquid interface (LLI) and air-liquid interface (ALI). Means ± SD, n = 4/group. Statistical analysis: 2-way ANOVA and Bonferroni test. ****P*<0.001 compared to the LLI groups.

Morphological analysis of the CFBE cultures by immunostaining of the junctional proteins ZO-1 and E-cadherin confirmed the damaged cell layer integrity (Figure S2). More apoptotic cell nuclei and loss of junctional staining pattern was observed in case of the ALI-cultured cells.

**
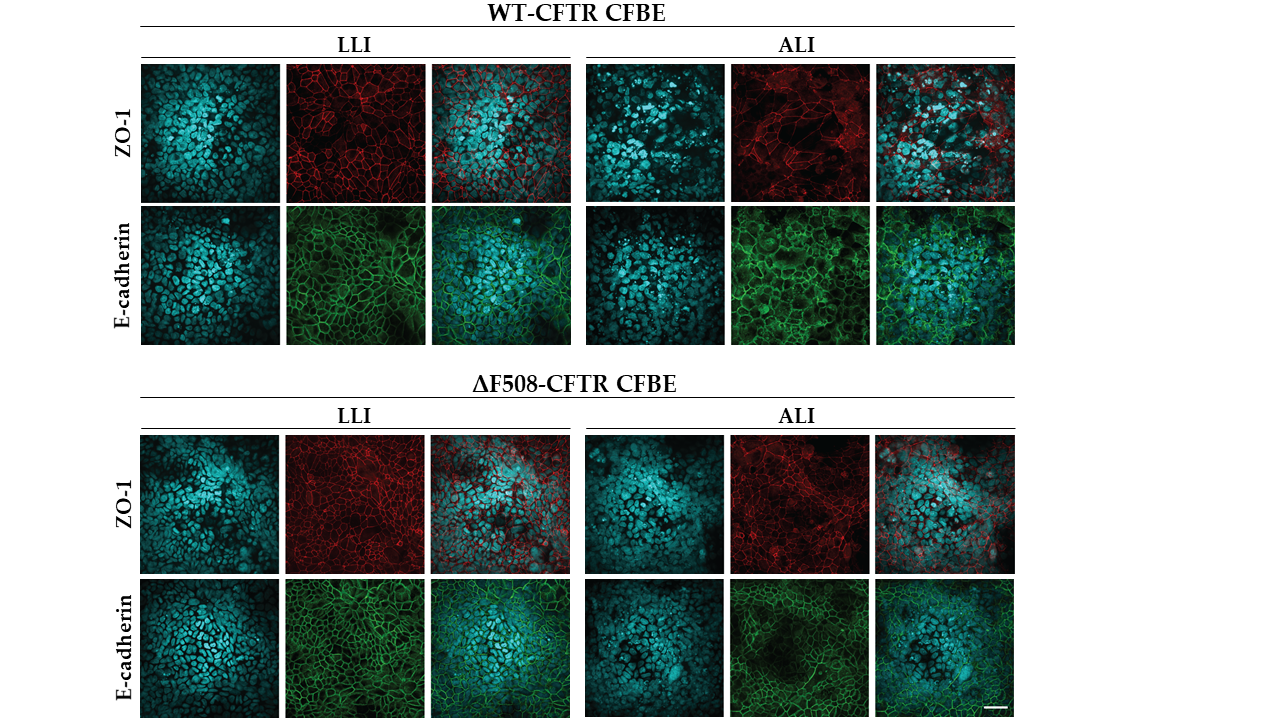
**

**Figure S2.** Immunostaining of junctional proteins ZO-1 and E-cadherin in CFBE cells cultured at liquid-liquid interface (LLI) and air- liquid interface (ALI). Green and red color: immunostaining for junctional proteins. Cyan color: staining of cell nuclei. Bar: 40 µm.

**2. The effect of cytokines on the barrier integrity of CFBE monocultures**

The 6-hour treatment with TNFα (50 ng/ml) and IL-1β (25 ng/ml) cytokines did not alter significantly the TEER values (Figure S3A) or permeability of CFBE monocultures for albumin (Figure S3B). The changes in the permeability values of WT-CFTR CFBE cells for the small hydrophilic marker fluorescein were minimal, especially compared to changes seen in the co-culture models (Figure 4). It is important to note that both cell lines formed tight barriers which were reflected in the very low permeability values (P_app_ for fluorescein: 0.30 ± 0.02 × 10^-6^ cm/s).


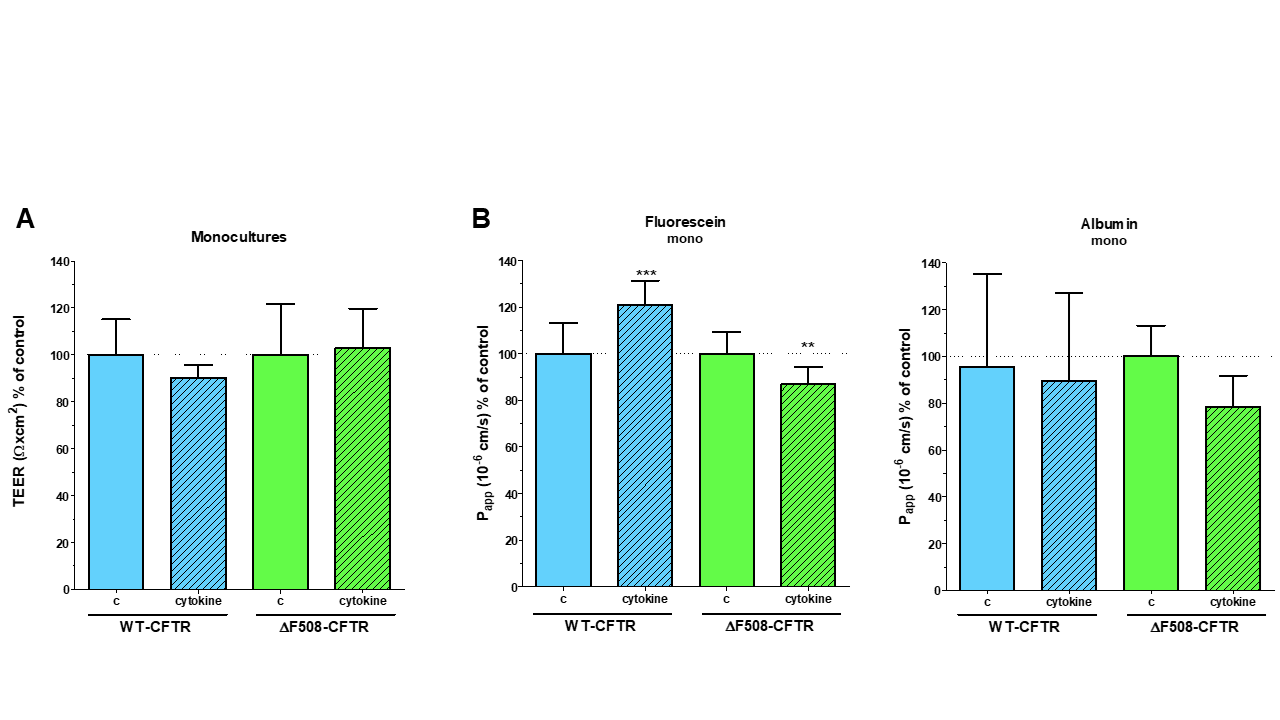


**Figure S3.** Transepithelial electrical resistance (A) and permeability values (B) of monocultures after 6-hour cytokine treatment. Values are presented in the percentage of control groups. Means ± SD, n = 4/group. Statistical analysis: 2-way ANOVA followed by Bonferroni test. ***P*<0.01, ****P*<0.001 compared to the control groups.

**3. The effect of sodium bicarbonate on CFBE cell viability**

After 1-hour bicarbonate treatment only the highest, 200 mM concentration decreased the impedance of CFBE cells (Figure S4A). After 24 hours the cell of index of WT-CFTR CFBE cells was lower in the 50 and 100 mM bicarbonate treatment groups (Figure S4B) suggesting that cells expressing wild type channels are more sensitive to bicarbonate. In ΔF508-CFTR CFBE cells the same cell index values were measured in the control and the 50 and 100 mM bicarbonate treatment groups at both short and long term time points which indicate a beneficial effect of the bicarbonate.


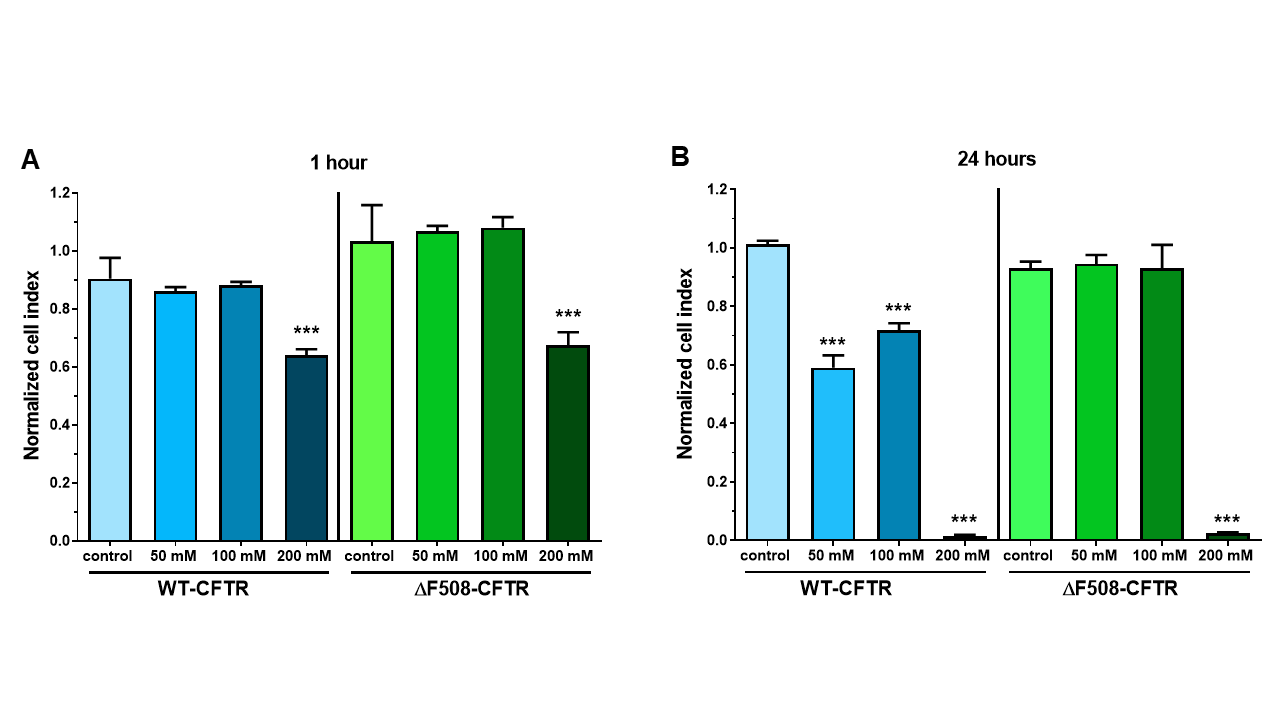


**Figure S4.** Impedance measurements of WT-CFTR CFBE (A) and ΔF508-CFTR CFBE (B) cells after treatment with culture medium containing different concentrations of sodium bicarbonate (50, 100, 200 mM). Culture medium in the control groups contains 26 mM sodium bicarbonate. The effects of bicarbonate on the impedance were shown as normalized cell index. Values are presented as means ± SD, n = 11-13. Statistical analysis: ANOVA and Bonferroni test. ****P*<0.001 compared to the control groups.

**4. The effect of bicarbonate on the barrier integrity of CFBE monocultures**

TEER values of the CFBE cell layers decreased after 1-hour bicarbonate treatment (WT-CFTR: 57% of the control, ΔF508-CFTR: 73% of the control), but the resistance values returned to the level of the control group in WT-CFTR CFBE cells by 24 hours (Figure S5A). In case of ΔF508-CFTR CFBE cells resistance decreased further and at the 24-hour time point it was 62% of the TEER in the control group. Permeability values remained fairly constant for both tracers in all groups indicating tight barrier properties, except ΔF508-CFTR CFBE cells where 1.5 times higher fluorescein permeability could be observed after 24-hour treatment. These data support our observation that sodium bicarbonate at 100 mM concentration does not disturb the barrier properties of CFBE cells.


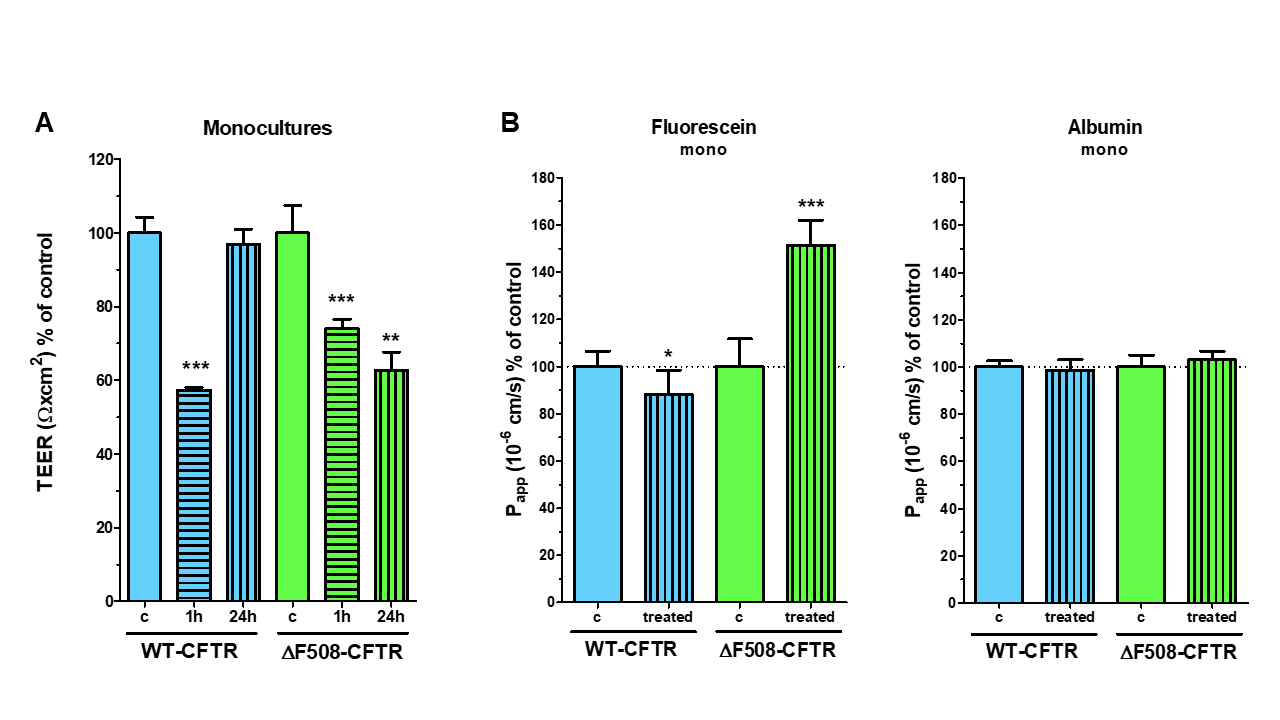


**Figure S5.** The effect of sodium bicarbonate on the barrier integrity of CFBE cells in monoculture. Transepithelial electrical resistance values at 1 and 24- hour time points (A) and permeability values (B) of the monoculture models after treatment with culture medium containing 100 mM sodium bicarbonate. Values are presented in the percentage of control groups. Means ± SD, n = 4/group. Statistical analysis: 2-way ANOVA followed by Bonferroni test. **P*<0.05, ***P*<0.01, ****P*<0.001****P*<0.001 compared to the control groups.

In concordance with data obtained on co-cultures (Figure 10) the 24-hour bicarbonate treatment did not visibly change the staining pattern of the sodium bicarbonate-treated groups in CFBE bronchial epithelial cells in monoculture (Figure S6). A tight barrier was visualized by the localization of the junctional proteins, cell-cell connections formed pericellular belts in the control groups. No major morphological change could be observed for the treatment groups.


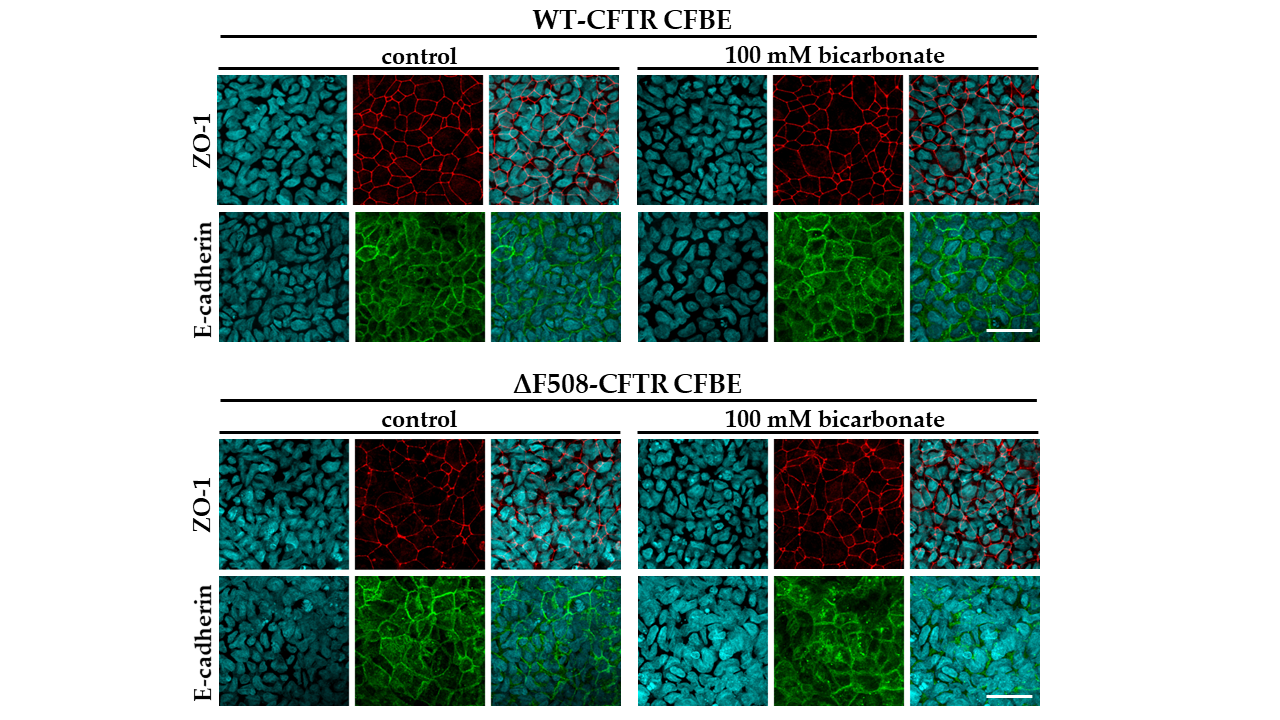


**Figure S6.** The effect of sodium bicarbonate on the junctional morphology of CFBE cells in monoculture. Immunostaining of the monoculture models for junction proteins zonula occludens-1 (ZO-1) and E-cadherin after treatment with culture medium containing 26 mM (control group) or 100 mM bicarbonate treatment (24 h). Red color: immunostaining for ZO-1. Green color: immunostaining for E-cadherin. Cyan color: cell nuclei. Bar: 40 µm.
