# Supplementary figures and images for: The Effect of Sodium Bicarbonate, a Beneficial Adjuvant Molecule in Cystic Fibrosis, on Bronchial Epithelial Cells Expressing a Wild-Type or Mutant CFTR Channel
Source: Int J Mol Sci. 2020 Jun 4;21(11):4024. doi: 10.3390/ijms21114024 (PMC7312297; doi:10.3390/ijms21114024)

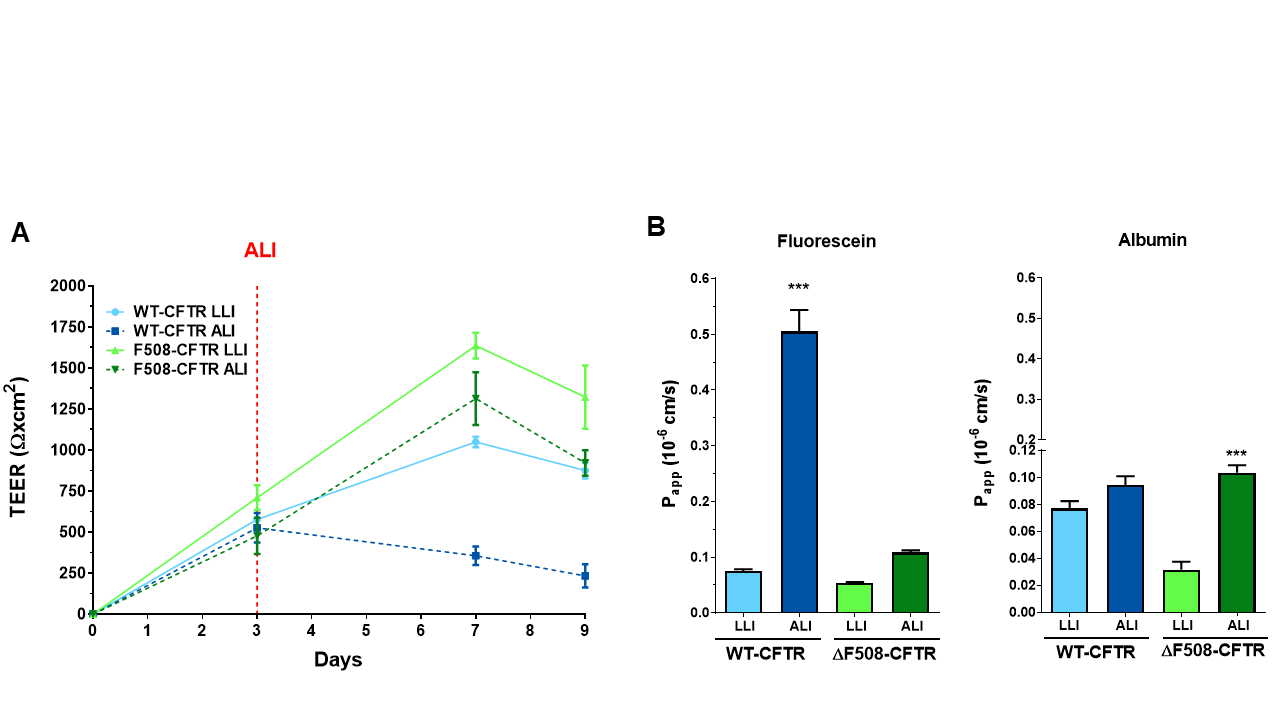

Supplement: Supplementary file 1 [file ijms-21-04024-s001.zip › Supplementary/Suppl. Figure 1..tif]

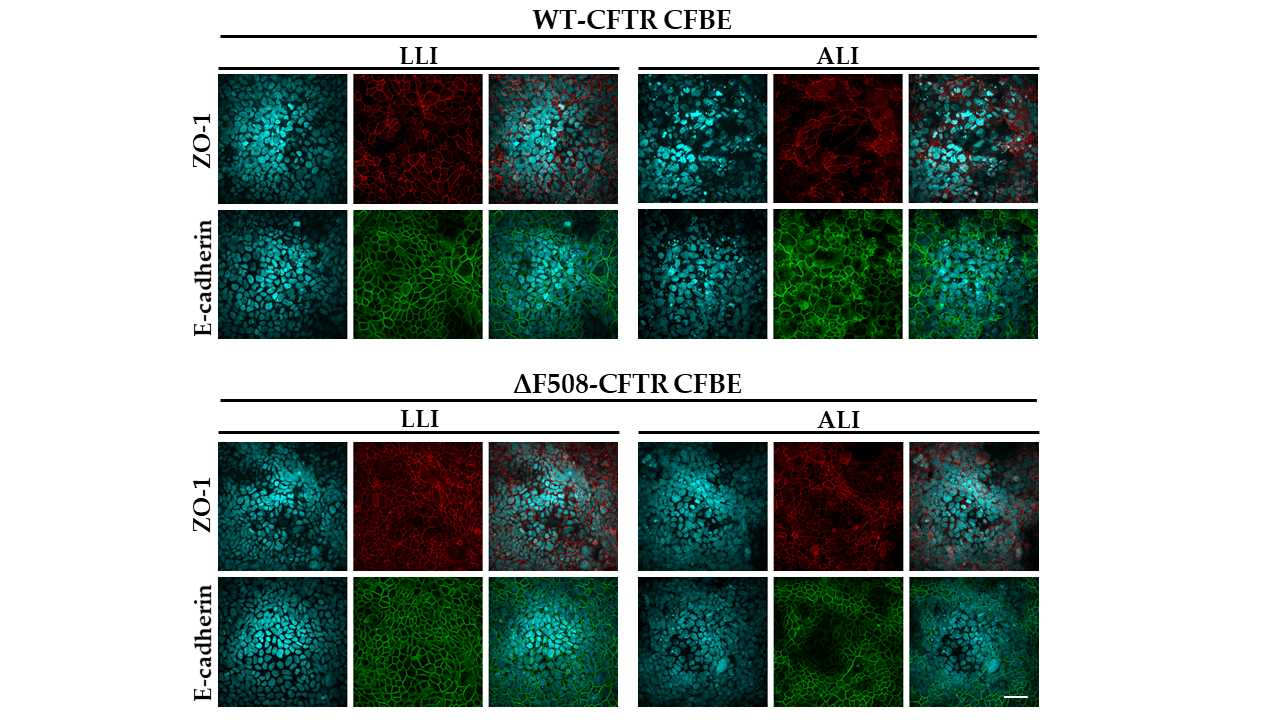

Supplement: Supplementary file 1 [file ijms-21-04024-s001.zip › Supplementary/Suppl. Figure 2..tif]

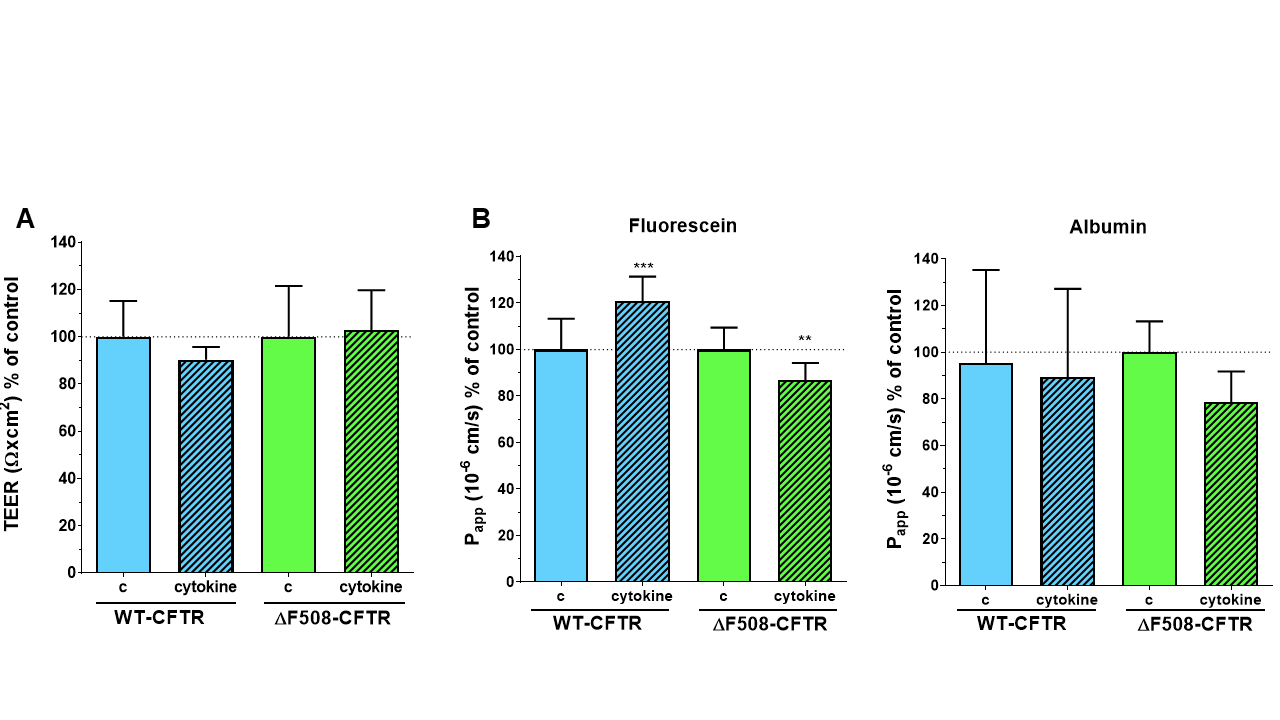

Supplement: Supplementary file 1 [file ijms-21-04024-s001.zip › Supplementary/Suppl. Figure 3..tif]

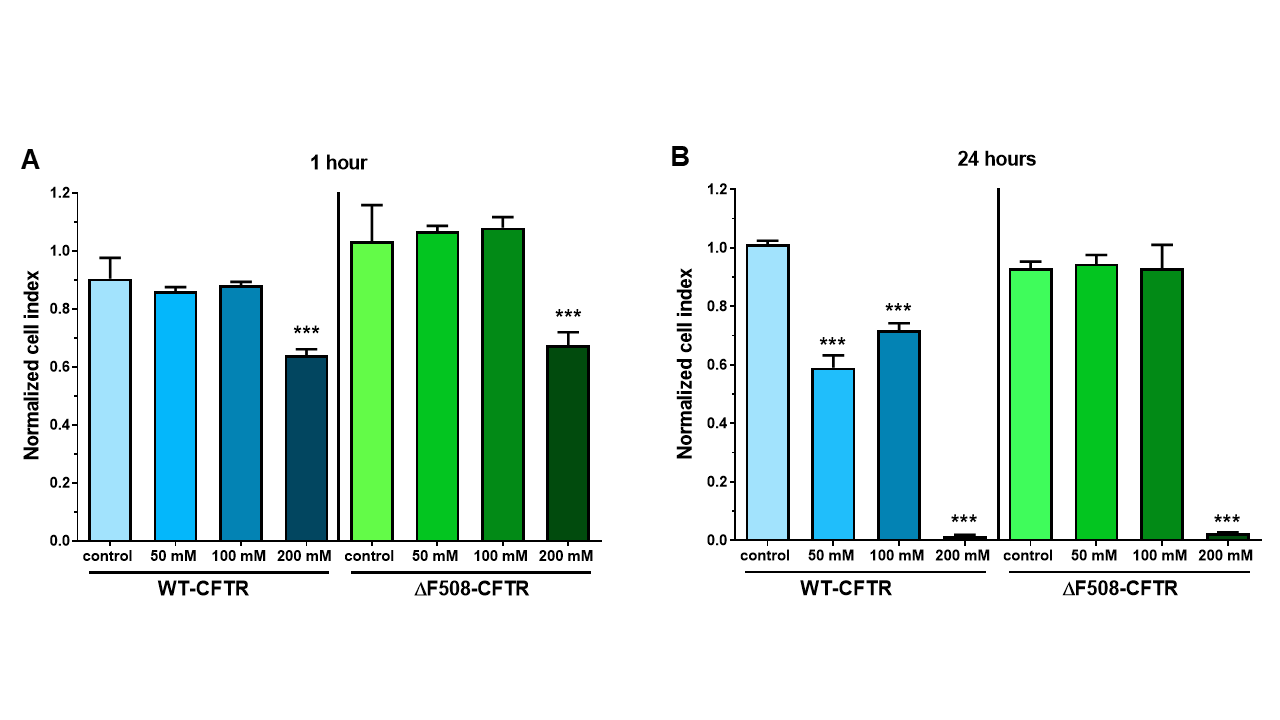

Supplement: Supplementary file 1 [file ijms-21-04024-s001.zip › Supplementary/Suppl. Figure 4..tif]

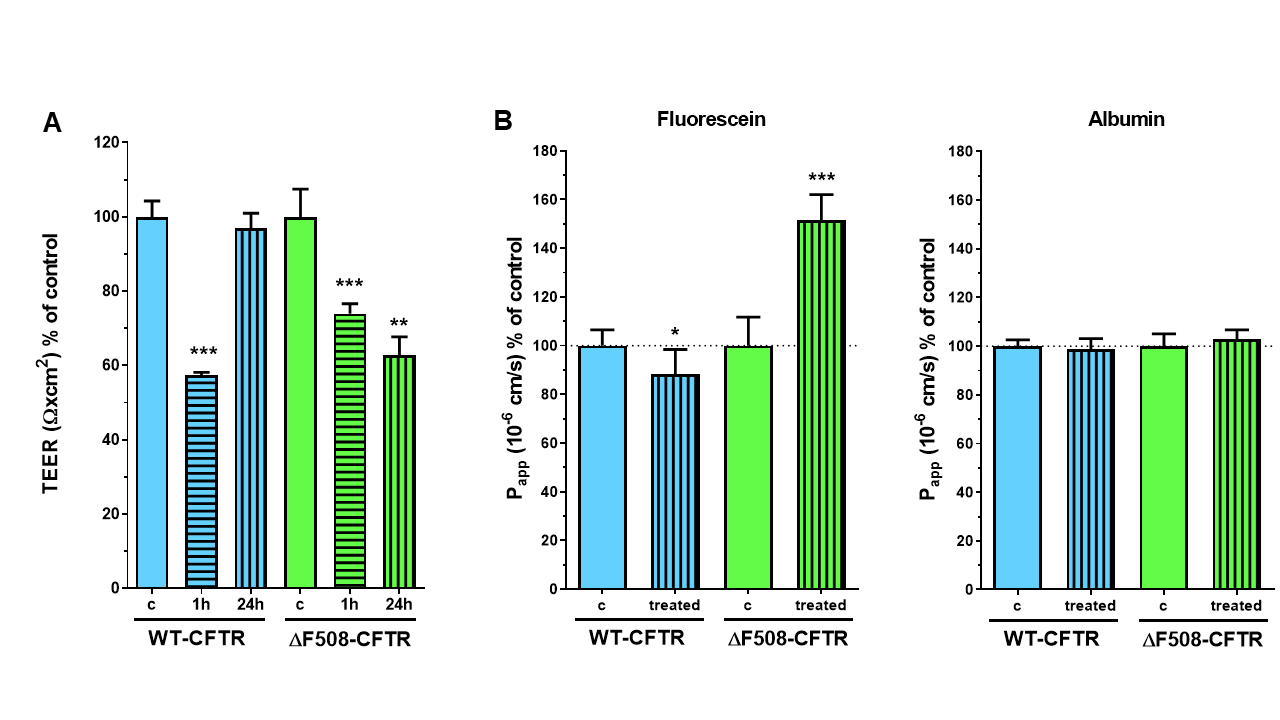

Supplement: Supplementary file 1 [file ijms-21-04024-s001.zip › Supplementary/Suppl. Figure 5..tif]

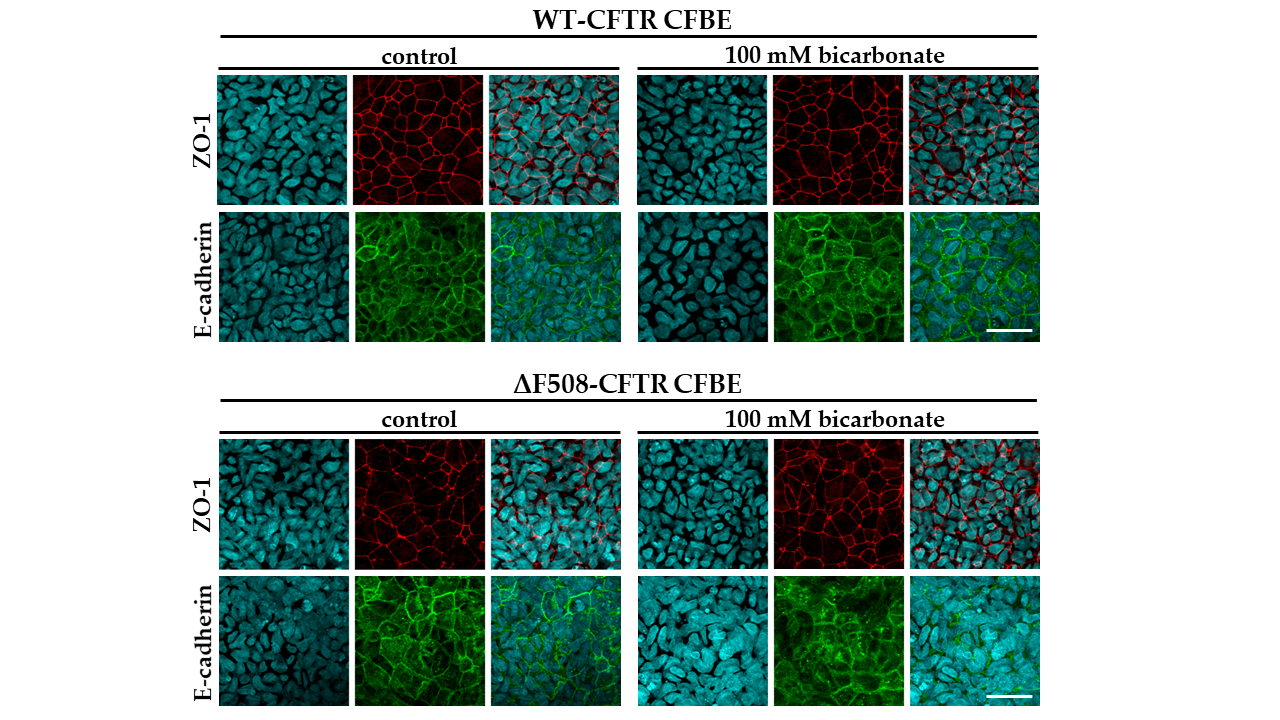

Supplement: Supplementary file 1 [file ijms-21-04024-s001.zip › Supplementary/Suppl. Figure 6..tif]
